# Pirfenidone Reverts Global DNA Hypomethylation, Promoting DNMT1/UHRF/PCNA Coupling Complex in Experimental Hepatocarcinoma

**DOI:** 10.3390/cells13121013

**Published:** 2024-06-10

**Authors:** Hipolito Otoniel Miranda-Roblero, Liliana Faridi Saavedra-Salazar, Marina Galicia-Moreno, Scarlet Arceo-Orozco, Fernando Caloca-Camarena, Ana Sandoval-Rodriguez, Jesús García-Bañuelos, Claudia Frias-Gonzalez, Mónica Almeida-López, Erika Martínez-López, Juan Armendariz-Borunda, Hugo Christian Monroy-Ramirez

**Affiliations:** 1 Programa de Doctorado en Ciencias en Biología Molecular en Medicina, CUCS, University of Guadalajara, Guadalajara 44340, Mexico; hipo.otto@gmail.com (H.O.M.-R.); liliana.saavedra@alumnos.udg.mx (L.F.S.-S.);; 2 Institute of Molecular Biology in Medicine and Gene Therapy, Department of Molecular Biology and Genomics, University Center of Health Sciences, University of Guadalajara, Guadalajara 44100, Mexico; marina.galicia@academicos.udg.mx (M.G.-M.); arceorozco93@gmail.com (S.A.-O.); fernando.caloca@alumnos.udg.mx (F.C.-C.); anasol44@hotmail.com (A.S.-R.); jesus.gbanuelos@academicos.udg.mx (J.G.-B.); 3 University Center of Health Sciences, University of Guadalajara, Guadalajara 44340, Mexico; 4 Institute of Translational Nutrigenetics and Nutrigenomics, Department of Molecular Biology and Genomics, University Center of Health Sciences, University of Guadalajara, Guadalajara 44100, Mexico; 5 Tecnologico de Monterrey, School of Medicine and Health Sciences, Zapopan 45138, Mexico

**Keywords:** hepatocellular carcinoma, DNMT1, DNMT3a, c-Myc, β-catenin

## Abstract

Hepatocellular carcinoma (HCC) development is associated with altered modifications in DNA methylation, changing transcriptional regulation. Emerging evidence indicates that DNA methyltransferase 1 (DNMT1) plays a key role in the carcinogenesis process. This study aimed to investigate how pirfenidone (PFD) modifies this pathway and the effect generated by the association between c-Myc expression and DNMT1 activation. Rats F344 were used for HCC development using 50 mg/kg of diethylnitrosamine (DEN) and 25 mg/kg of 2-Acetylaminofluorene (2-AAF). The HCC/PFD group received simultaneous doses of 300 mg/kg of PFD. All treatments lasted 12 weeks. On the other hand, HepG2 cells were used to evaluate the effects of PFD in restoring DNA methylation in the presence of the inhibitor 5-Aza. Histopathological, biochemical, immunohistochemical, and western blot analysis were carried out and our findings showed that PFD treatment reduced the amount and size of tumors along with decreased Glipican-3, β-catenin, and c-Myc expression in nuclear fractions. Also, this treatment improved lipid metabolism by modulating PPARγ and SREBP1 signaling. Interestingly, PFD augmented DNMT1 and DNMT3a protein expression, which restores global methylation, both in our in vivo and in vitro models. In conclusion, our results suggest that PFD could slow down HCC development by controlling DNA methylation.

## 1. Introduction

Hepatocellular carcinoma (HCC) represents the most frequent primary liver cancer and the third leading cause of death related to this illness worldwide [1]. Several etiological factors are associated with HCC genesis, i.e., HBV and HCV viral infection, diseases linked with metabolic disorders, and excessive alcohol intake, which are amongst the most frequent [2]. On the other hand, HCC development is associated with an increase in oxidative, inflammatory, fibrogenic, and proliferative events and an increase in growth and cellular death [3]. Mutations in specific genes involved in these processes mediate many of these processes. In recent years, interest has grown in studying the effects of the environment on the expression of critical genes involved in the genesis of various diseases, including cancer. More importantly, understanding the expression of a given gene when altered and without modifying its nucleotide sequence has become a key issue in this new era of pharmaceutics and epigenomics [4].

Epigenetic modifications depend on environmental factors that favor changes in a particular gene expression, while no alterations in its DNA sequence occur [5]. Two types of epigenetic modifications related to the initiation and development of cancer are known: DNA methylation and histone modifications. DNA methylation mainly occurs on the cytosine ring, specifically CpG islands, driven by DNA methyltransferases (DNMTs) activity. Global hypomethylation of the genome is a common feature in many cancers, including HCC. This widespread hypomethylation can contribute to genomic instability and the activation of other oncogenes. Primarily, methylation of the gene promoter region causes transcriptional repression, while methylation in the gene body promotes gene expression in various cancers, including HCC [4].

On the other hand, experimental models are an adequate strategy for evaluating the efficacy and safety of new drugs [6]. Castro-Gil et al. used an HCC animal model administering diethylnitrosamine (DEN) and 2-acetylaminofluorene (2AAF) to rats. This method limited weight gain and increased hepatomegaly and liver changes. Also, increased levels of liver cancer markers like γ-GTP, prostaglandin reductase 1, and GSTP1 were found. Importantly, this experimental model allowed the study of HCC development from its earliest stages to its final stage [7].

Pirfenidone (PFD) is a drug with important pharmacological properties; evidence from basic and clinical studies has shown that PFD has antifibrotic, antioxidant, and anti-inflammatory effects [8,9]. In in vitro models, PFD can inhibit proliferation, promote apoptosis of HepG2 cells [10], and regulate SIRT1-related mechanisms [11]. Silva-Gómez et al. demonstrated that PFD efficacy is involved in preventing HCC genesis via induction of p50 nuclear translocation, modifying the p65/p50 ratio in favor of p50, and knocking down IL-6, TNF-α, and COX-2 expression. In the early stages of experimental HCC, PFD also changed the expression of p53, the activation of caspase-3p17, and the cleavage of PARP-1 [12].

Previous studies have reported that expression levels of oncogenes such as c-Myc, cyclinD1, ß-catenin, and tumor suppressor genes such as p53, E-cadherin, DLC-1, and pRb are downregulated to different degrees during the development of HCC. Particularly, c-Myc is associated with hypomethylation, while p53 has been associated with hypermethylation in its promoter sequence [13].

Our main goal in this study was to elucidate whether PFD regulates the formation of DNMT1/3a complexes and restores global DNA methylation, which might be related to HCC progression.

## 2. Material and Methods

### 2.1. Experimental Animals

Male Fischer-344 rats were provided by UPEA-Bioterio at CUCS, “Universidad de Guadalajara”, México and housed in accordance with the guidelines of Universidad de Guadalajara under the approval number of the bioethics and research committees CI-03020. Animals were housed under a 12-h light/dark cycle and constant temperature of 25 °C ± 2 °C and received humane care. 

### 2.2. Animals and Experimental Design

**Figure 1:**
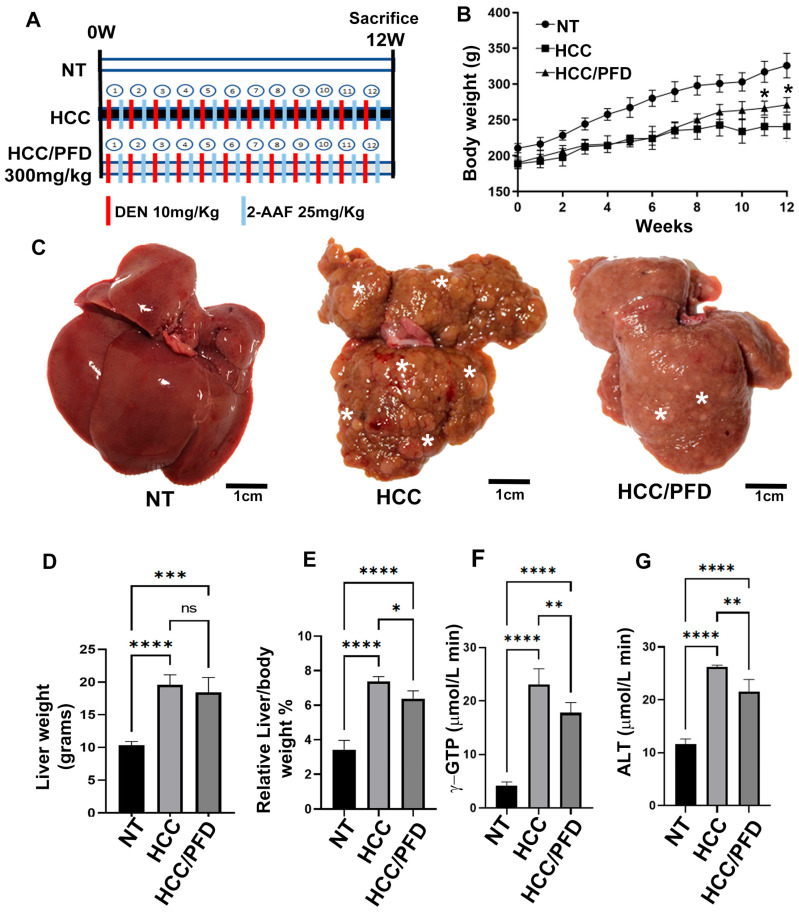
Liver damage caused by chemicals is prevented by PFD. (**A**) Established experimental design; NT, non-treated group; HCC, hepatocellular carcinoma group injected weekly with DEN (50 mg/kg/i.p.) plus 2AAF (25 mg/kg/p.o.); HCC/PFD group, HCC treatment plus 300 mg/kg PFD from week 0. All experimental groups were euthanized after 12 weeks. (**B**) Weekly logging of body weight. (**C**) Representative images of livers after 12 weeks of treatment. Greater size and number of dysplastic nodules were observed in HCC group than in the HCC/PFD group (white asterisks). (**D**) Graph of the liver weight at the end of treatment. (**E**) The ratio of liver weight to body weight of animals in each study group. (**F**) Serum gamma-glutamyl transferase (GTP) assay. (**G**) Serum alanine transaminase (ALT) assay. Data are presented as mean ± SD using ANOVA followed by Tukey’s multiple comparison test. ns, not significantly different, * *p* < 0.05, ** *p* < 0.01, *** *p* < 0.001, **** *p* < 0.0001.

A hepatocarcinogenesis model implemented by Castro-Gil et al. [7] was developed. Eighteen rats of the same weight (180 g) were randomized into three groups: No treatment (NT, n = 6), administered with the vehicle (0.5% carboxymethyl cellulose, p.o. (CMC); hepatocellular carcinoma (HCC, n = 6); rats were injected weekly with DEN (50 mg/kg/i.p. Sigma-Aldrich, St. Louis, MO, USA) plus 2AAF (25 mg/kg/p.o. Sigma-Aldrich); and HCC/PFD group (n = 6), administered with the same treatments of HCC group, plus PFD 300 mg/kg/day, p.o. (Tecoland, Irvine, CA, USA). Noteworthy, this protocol was carried out for 12 weeks (Figure 1A) instead of 4 weeks as previously described (11). Finally, all animals were euthanized by administering isoflurane (PISA, Guadalajara, Jalisco, Mexico). 

### 2.3. Biochemical Determination of γ-GTP and ALT

Alanine aminotransferase (ALT) and gamma-glutamyl transpeptidase γ-GTP) activities were measured in rat plasma as described in Supplementary Material [12].

### 2.4. Histologic Assessment of Liver Sections

Liver samples from all animals were fixed with 10% formaldehyde for 24 h. They were subsequently embedded in paraffin. Sections were sliced into 4 μm-thick pieces and mounted in glass slides. For the different stains performed, tissues were deparaffinized at 60 °C overnight in xylene and hydrated in graded ethanol solutions. HCC diagnosis was histologically confirmed by Glypican-3 (GPC3, GENETEX, Irvine, CA, USA), and all HCC tumor tissues were assessed by hematoxylin and eosin staining. Fibrosis was evaluated with Masson trichrome. A certified pathologist analyzed liver histology. All experiments were performed according to standard procedures.

### 2.5. Cell Culture and Treatments

To perform in vitro assays, HepG2 cells (ATCC. HB-8065, Manassas, VA, USA) were grown in Dulbecco’s modified Eagle medium (DEMEM; Invitrogen Life Technologies-GIBCO, Carlsbad, CA, USA) supplemented with 10% fetal bovine serum (FBS; Invitrogen Life Technologies-GIBCO), 100 U/mL penicillin, and 100 g/mL streptomycin (GIBCO. Life Technologies) in a humidified atmosphere of 5% CO_2_ in air at 37 °C. Cells were seeded at a density of 3 × 10^5^ cells per mL. Treatments were performed after 8 h of fetal bovine serum starving. Then, cells were incubated with the inhibitor of DNMTs, 5-aza-2′-deoxycytidine (5-Aza) (0.05 mM) (Santa Cruz Biotechnology, Dallas, TX, USA), Rosiglitazone (Ros) (0.03 mM) (Santa Cruz Biotechnology, Dallas, TX, USA), and PFD (0.5 mM) for 48 h to evaluate effect on DNMTs and proteins expression (See Supplementary Table S1). DMSO (0.1 μM) was used as control.

### 2.6. Proteins Extraction and Western Blot Assay

Samples of normal and HCC tissue or HepG2 cell cultures were extracted using nuclear and cytoplasmic extraction reagents, containing a protease inhibitor and a phosphatase inhibitor cocktail (Thermo Fisher Scientific, Waltham, MA, USA). The extracts were collected and centrifuged at 17,000 rpm at 4 °C for 10 min (GYROZEN 1730MR, Gimpo, Korea). Mini-Bradford determined protein concentrations. Proteins were boiled for 10 min in Laemmli Sample Buffer 2X (Bio-Rad, Hercules, CA, USA), separated with SDS-PAGE, and transferred to PVDF membranes (Bio-Rad). The membranes were blocked with 10% non-fat milk in phosphate-buffered saline (PBS) containing 0.1% Tween-20 (Bio-Rad) at 4 °C overnight. Multiple membranes were probed with primary antibodies (see Supplementary Table S1) overnight at 4 °C and then incubated with secondary antibodies for 2 h at room temperature. Bands of interest were visualized using BioRad ChemiDoc™ XRS+ System software ImageLab 5.2.1 (Bio-Rad).

### 2.7. Immunofluorescence

Immunofluorescence for DNMT1, DNMT3a, DNMT3b, and 5mC in tissue and HepG2 cells was performed as described by Silva-Gomez et al. [11]. Nuclei were stained with 4′,6-diamidino-2-phenylindole (DAPI) (Sigma-Aldrich). Images were collected using an epifluorescence microscope OLYMPUS BX51 (Shinjuku, Tokyo, Japan) and analyzed using Image-ProPlus 6.0 (OLYMPUS, Shinjuku, Tokyo, Japan).

### 2.8. Global Methylation Assessment in Genomic DNA

Total DNA was extracted from liver tissue using the commercial kit QIAamp DNA Mini Kit (Qiagen, Hilden, Germany) following provider instructions. DNA was quantified using NanoDrop, and 100 ng of DNA was used to measure the percentage of total methylation using MethylFlash Global DNA Methylation (5mC) ELISA (EpiGentek, Farmingdale, NY, USA) and compared against a standard curve. Briefly, wells were pretreated to generate a high affinity for DNA. Subsequently, a capture antibody (5mC) specific against 5-methylcytosine was included. Finally, a developing solution that binds to the capture antibody was added, allowing colorimetric detection at 450 nm. The amount of methylated DNA was proportional to the intensity of the optical density obtained, and compared to the calibration curve using the following formula:

5mC% = (sample od − negative control od)/(slope × ng DNA) × 100%

### 2.9. Dot Blot of Global DNA Methylation

Dot blot was used to determine global DNA methylation through 5mC detection in cells and liver tissue. DNA was denatured by incubation with 0.1 M of NaOH for 10 min at 95 °C; to prevent annealing, DNA was kept on ice. DNA solution was neutralized by 1 M of NH4OAc for 1 min. A total of 30 µg in 3 µL of DNA was dot-blotted for 30 min at 80 °C in a nitrocellulose membrane (Bio-Rad). The membranes were blocked with 5% BSA (Sigma-Aldrich) for 1 h/TBS-T (Bio-Rad) at room temperature and were incubated with a mouse anti-5mC monoclonal antibody and IgG antibody as an isotype control. Finally, membranes were washed for 5 min 3 times in TBS-T and were visualized using Bio-Rad ChemiDoc™ XRS+ System software ImageLab 5.2.1. 

### 2.10. Protein–Protein Interaction Analysis through the STRING Platform

For the construction of the protein–protein interaction network, particularly between PPARγ and DNMTs, the STRING tool v11.5 (https://string-db.org/ (accessed on 25 January 2024)) was used. This tool allows the integration of all known possible IPPs. The information of proteins to be studied is introduced to the server, STRING searches for all interactions between the corresponding molecules and generates a network with nodes (proteins) and edges (interaction).

### 2.11. Molecular Docking Protein–Protein

A crystal structure of both proteins was downloaded from Protein Data Bank (https://www.rcsb.org/ (accessed on 28 May 2024)). We examined two variants of PPARγ to identify which specific PPARγ domain facilitates the formation of the complex with DNMT1. The two structures mentioned are 3QT0, which contains the LBD domain, and 3DZY, which contains the DBD domain in conjunction with RXR). Subsequently, to predict the possible interaction between both proteins, the PDB codes of each protein were entered into the HawkDock server to perform molecular docking. The HawkDock server determines the best positions and protein–protein docking sites through HawkRank and ATTRAC, and MM/GBSA allows the identification of the key residues that determine the interaction [14].

### 2.12. Statistical Analysis

All data is expressed as the mean values ± SD. ANOVA followed by Tukey’s was used to test statistical significance between groups as appropriate. Graphs and statistical analysis were generated using GraphPad Prism 10.0 software (GraphPad Software, San Diego, CA, USA). Differences were considered statistically significant when *p* < 0.05.

All assays and analyses were performed in triplicate, as shown in Supplementary Figures S2–S12. 

## 3. Results

### 3.1. Liver Damage Caused by Chemicals Is Prevented by Pirfenidone

Carcinogenic damage induced during twelve weeks by the administration of DEN and 2-AAF caused a decrease in body weight in animals of HCC group (Figure 1B); in addition, in this same group, severe morphological alterations in liver tissue and neoplastic nodules development were observed (Figure 1C). Also, hepatomegaly observed in HCC group was corroborated by quantifying the liver weight/body weight ratio, which increased significantly in damage group rats versus NT group (*p* < 0.0001) (Figure 1D,E). Regarding serum markers of liver damage, γ-GTP and ALT, activity of both enzymes increased significantly, corroborating the deterioration of liver function induced in the HCC group (Figure 1F,G). Noteworthy, the concomitant administration of PFD favored the recovery of body weight from the eighth week of the experimental procedure, prevented the macroscopic and microscopic alterations observed in the damaged group, and prevented the increase in the activity of liver enzymes.

All data of the morphological evaluation of liver tissue are shown in Table 1 and Table 2. It is important to mention that in HCC group livers, as well as in livers of rats administered with PFD, the formation of nodules is observed; however, in the HCC/PFD group, nodule number and their size was significantly lower than in the HCC group (less than 1 mm (*)), Figure 1C.

### 3.2. Pirfenidone Slows down the Expression of Carcinogenic Markers, Reduces Fibrosis, and Decreases Damage in Liver Architecture

**Figure 2:**
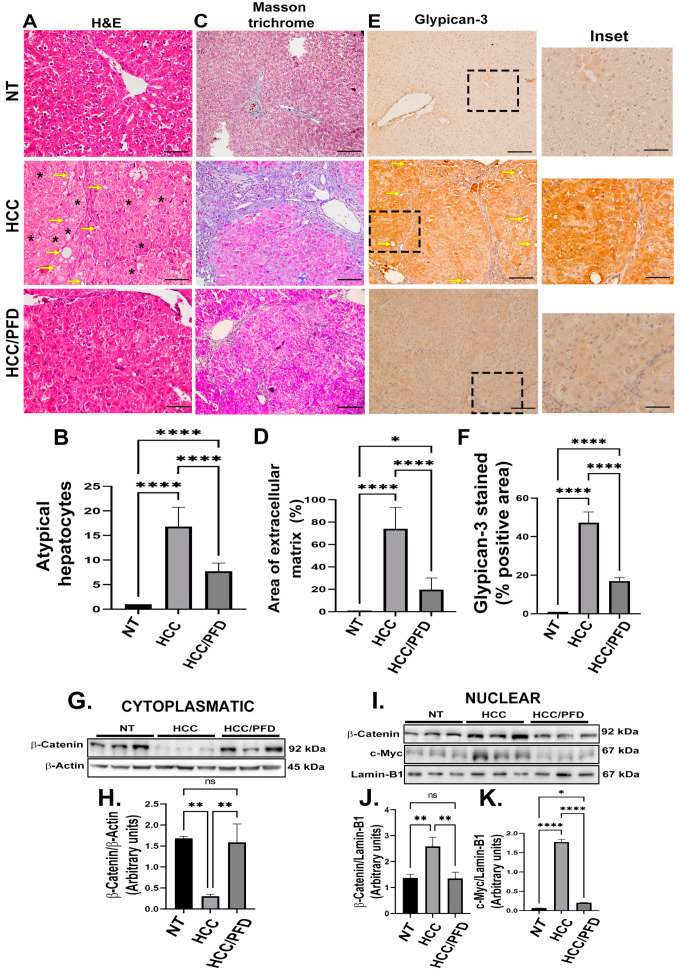
PFD prevented alteration of hepatic architecture, fibrosis, and neoplastic lesions. (**A**) Photomicrograph representative of H&E staining (H&E) of groups at 12 weeks. Deformed portal tracts and thickened hepatic plaques are evident in HCC (asterisks). (**B**) Quantification of atypical hepatocytes. Cells with many nuclei and changes in the nuclear–cytoplasmic ratio (asterisk), in addition to numerous steatosis sites (yellow arrows). (**C**) Masson’s trichrome staining (MT). (**D**) Quantification of the percentage of collagen fibers deposited in the liver tissues of the different groups. (**E**) Representative expression of GPC3. Hepatic GPC-3 was analyzed in tissue by immunohistochemistry using primary anti-rabbit GPC-3 antibody. (**F**) Percentage of areas positive for GPC-3. (**G**) Representative western blot of cytoplasmic β-catenin. (**H**) Quantification of β-catenin expression. (**I**) Western blotting representative of the nuclear fraction of β-catenin and c-Myc. (**J**) Quantification of β-catenin nuclear expression. (**K**) Quantification of c-Myc nuclear expression. Significantly different at * *p* < 0.05, ** *p* < 0.001, **** *p* < 0.00001. ns, not significantly different.

To demonstrate the PFD effect on microscopic alterations in liver parenchyma, H&E and Masson’s trichrome stains were performed. Additionally, IHC examined GPC3 as a marker of liver neoplastic lesions. Figure 2A shows microscopic changes in the HCC group suggestive of malignancy, such as abundant anaplastic hepatocytes, hyperchromic nuclei, and multinucleated cells with alterations in the nuclear–cytoplasmic ratio (asterisk), in addition to abundant foci of steatosis (yellow arrows) and a marked appearance of ductular reaction in the portal vasculature. On the other hand, PFD administration prevented these histological changes.

Moreover, in Figure 2C, extensive areas of fibrosis (positive turquoise blue) in the liver of rats with HCC were observed. In addition, greater sinusoidal capillarization and a high infiltration of inflammatory cells were detected. On the contrary, the administration of PFD prevented the accumulation of extracellular matrix by 20% versus damage group (Figure 2D; *p* < 0.001) and averted inflammatory cells infiltration. Figure 2G shows the IHC for GPC3 detection in liver tissues from HCC group where the expression of this surface oncoprotein has significantly increased. Remarkably, PFD prevented the expression of GPC3 in approximately 50% (Figure 2F, *p* < 0.0001).

A cellular fractional process was performed to analyze the expression of cytoplasmic and nuclear proteins. Figure 2G displays representative western blots of the expression of β-catenin. A significant decrease in expression of this protein is observed in HCC rats compared to NT group, while the HCC/PFD group did not present significant changes (Figure 2H). On the other hand, in Figure 2I, it is observed that in the nuclear fraction, there is an increase in β-catenin and c-Myc in the HCC group; however, treatment with PFD significantly reduced the expression of both proteins in the cell nucleus (Figure 2J,K).

### 3.3. Pirfenidone Regulates Expression and Translocation of PPARγ and PPARγ2

**Figure 3:**
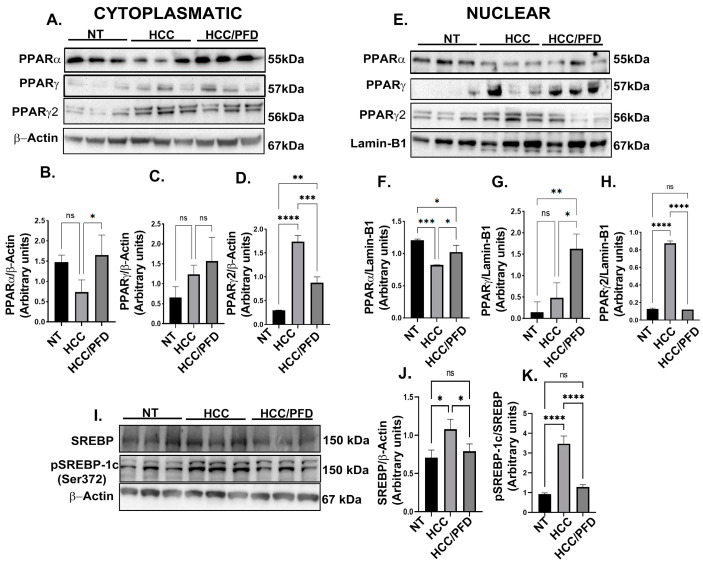
PFD modulates the expression and subcellular localization of PPAR isoforms and SREBP1. (**A**) Western blot representative of the cytoplasmic expression of different PPAR isoforms. (**B**) Densitometry determination of PPARα expression, (**C**) PPARγ and (**D**) PPARγ2. (**E**) Western blotting representative of the nuclear expression of different isoforms of PPARs. (**F**) Densitometry determination of the nuclear expression of PPARα (**G**) PPARγ and (**H**) PPARγ2. (**I**) Western blot representative of total and phosphorylated SREBP expression. (**J**) Graph of the determination of SREBP expression and (**K**) pSREBP-1c (ser372). The results are shown as the mean ± standard deviation (SD) of triplicate assays. One-way ANOVA and Tukey’s post-hoc tests were performed. Significantly different at * *p* < 0.05, ** *p* < 0.001, *** *p* < 0.0001 and **** *p* < 0.00001. ns, not significantly different.

To determine PPARα expression and localization, along with two isoforms of PPARγ, cytoplasmic and nuclear protein extracts were analyzed by western blot. Figure 3 shows that PFD significantly increases PPARα expression in both cytoplasm (3A and 3B; *p* < 0.05) and nuclear fraction (3E and 3F; *p* < 0.05), compared with the HCC group. In addition, this drug stimulates PPARγ expression only in the nuclear fraction (3E and 3G: *p* < 0.05) versus the damage group. Contrary to the above, PPARγ2 isoform expression exhibited a decrease in both cytoplasmic (3A and 3D; *p* < 0.0001) and nuclear fraction (3E and 3H: *p* < 0.00001) in groups treated with PFD compared with the HCC group. PPARγ2 is overexpressed in the liver and adipose tissue of animals developing fatty liver disease; it is also known that this transcription factor regulates SREBP1 protein function involved in the lipogenic response. Figure 3I–K shows that in the HCC group, the expression and phosphorylation of SREBP1 increased (*p* < 0.0001), specifically in serine 372; however, in the group treated with PFD, levels of this protein maintained their basal expression and phosphorylation.

### 3.4. Pirfenidone Reverses Global DNA Hypomethylation through Regulation of DNMT1

**Figure 4:**
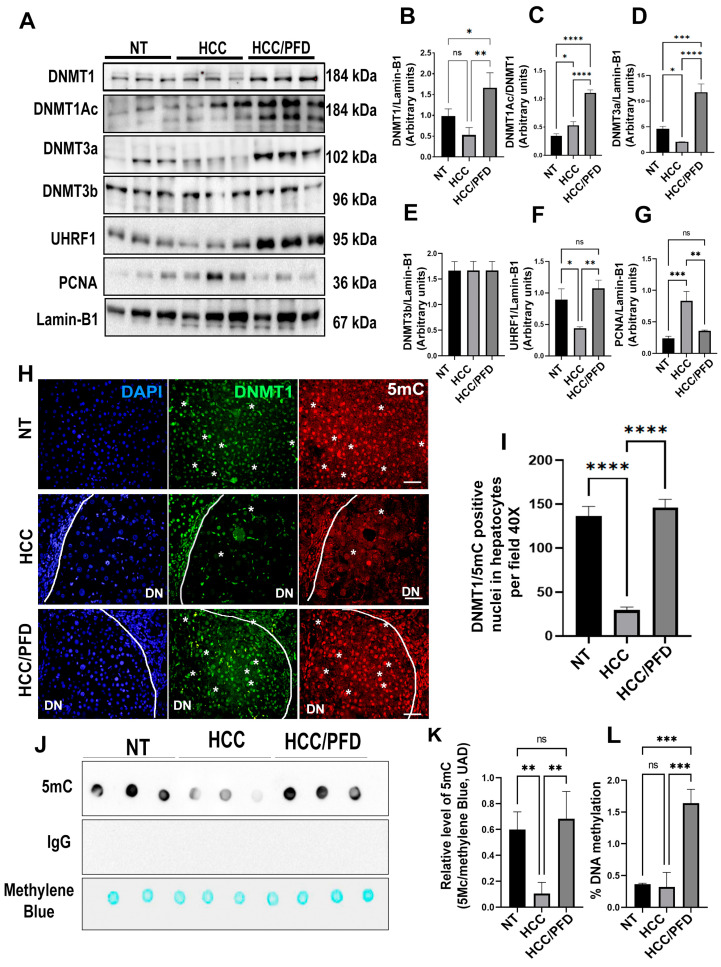
Pirfenidone modulates the expression of enzymes that modify DNA and promote global methylation. (**A**) Representative western blots of DNMT1, DNMT1Ac, DNMT3a, DNMT3b, UHRF1, and PCNA. (**B**) Graphs showing the relative expression levels of DNMT1, (**C**) DNMT1Ac, (**D**) DNMT3a, (**E**) DNMT3b, (**F**) UHRF1, and (**G**) PCNA. (**H**) Representative images of the nuclear localization of DNMT1 and 5mC in liver tissues. Nuclei were stained with DAPI (blue), DNMT1 (green) and 5-Methylcytosine (5mC) (red). Images were captured using an epifluorescence microscope. Dysplastic nodule (DN). White asterisks indicate positivity to the different markers analyzed. (**I**) Graph showing the number of positive hepatocytes for DNMT1. (**J**) Dot blot representative of global DNA methylation through the detection of 5mC. (**K**) Quantification of densitometry results of the relative levels of 5mC. (**L**) Determination of overall percentage of methylated DNA. One-way ANOVA and Tukey’s post-hoc tests were performed. Significantly different at * *p* < 0.05, ** *p* < 0.001, *** *p* < 0.0001, **** *p* < 0.00001. ns, not significantly different.

Nuclear extracts were used for the analysis of different DNMT isoforms, as well as for understanding of scaffold protein’s role, UHRF1, and PCNA. In our study, we analyzed PCNA as a clamp protein that facilitates DNMT1 function on hemimethylated DNA. Figure 4A shows representative western blots of the mentioned proteins. In HCC group, a decrease in DNMT1, DNMT3a, and UHRF1 nuclear expression, but not DNMT3b, is observed; in addition, a slight increase in DNMT1 acetylated form (DNMT1Ac; *p* < 0.05). However, in the PFD-treated group, a significant increase in DNMT1 expression (*p* < 0.001), DNMT3a (*p* < 0.0001), and UHRF1 (*p* < 0.0001), as well as DNMT1Ac (*p* < 0.00001) was observed. 

On the other hand, 5-methylcytosine (5mC) modification is the most frequent form of DNA methylation. To evaluate this mark, double immunofluorescence was carried out to detect the expression and localization of DNMT1 and modification of 5mC in liver tissue of experimental animals. In Figure 4H, the DNMT1 and 5mC signals present in the cellular nucleus of samples NT and HCC/PFD groups are observed. The first is distinguished in the green channel, while the second is in the red channel.

To corroborate the results obtained in the in vivo model, we carried out an in situ assay, analyzing the global DNA methylation by dot blot using a monoclonal antibody directed against 5mC, mouse IgG as isotype control and staining with methylene blue to control total DNA loading. Figure 4J,K, respectively, show that global DNA hypomethylation ensued in animals from HCC group. This response is prevented by PFD, which can maintain this global DNA methylation in a similar way to NT group (*p* < 0.001). Additionally, to corroborate the result obtained in the dot blot analysis, we performed an ELISA Methyl Flash Global DNA Methylation (5mC) assay. In Figure 4L, it was observed that in the HCC/PDF group, there was an increase in global DNA methylation versus the HCC group (*p* < 0.001).

### 3.5. Pirfenidone Regulates DNMT1 Expression and Prevents Global DNA Hypomethylation In Vitro

**Figure 5:**
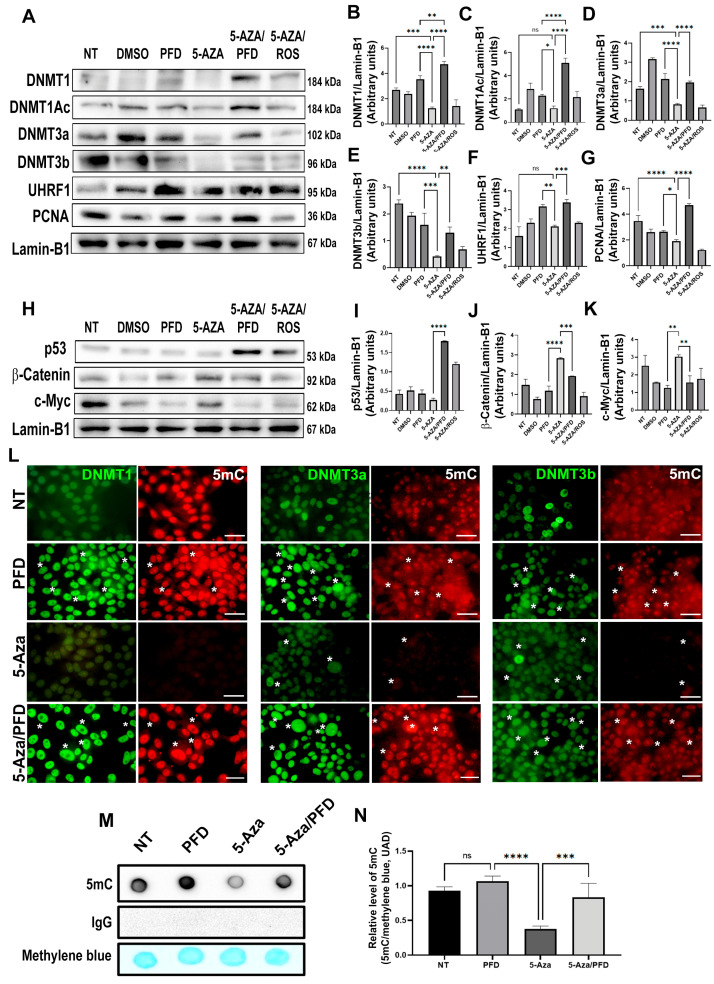
In vitro, pirfenidone affects the synthesis of enzymes altering DNA global methylation (**A**) Representative western blots for DNMT1, acDNMT1, DNMT3a, DNMT3b, UHRF1, and PCNA. Relative expression of DNMT1 (**B**), acDNMT1 (**C**), DNMT3a (**D**), DNMT3b (**E**), UHRF1 (**F**) and PCNA (**G**). Lamin-B1 was used as a loading control. (**H**) Representative western blots for p53, β-Catenin and c-Myc. Relative expression of p53 (**I**), β-Catenin (**J**) and c-Myc (**K**). Lamin-B1 was used as a loading control. (**L**) HepG2 cell nuclei were stained with DNMT1, DNMT3a and DNMT3B (green) and 5mC (red). White asterisks indicate positivity to the different markers analyzed. (**M**) Dot blot representative of global DNA methylation through 5mC detection. (**N**) quantification for 5mC relative levels, methylene blue was used as a DNA loading control. The results are shown as the mean ± standard deviation (SD) of triplicate assays. One-way ANOVA and Tukey’s post-hoc tests were performed. Significantly different at * *p* < 0.05, ** *p* < 0.001, *** *p* < 0.0001 and **** *p* < 0.00001. ns, not significantly different.

The HepG2 cell line was employed in in vitro studies to support the in vivo analyses. DNMT inhibitor, 5-Aza, and the PPARγ activator, rosiglitazone, were administered to cells for 48 h; subsequently, the nuclear extracts were purified and analyzed. Figure 5A–E demonstrate that 5-Aza administration reduces DNMT1, DNMT3a (*p* < 0.0001), and DNMT3b expression (*p* < 0.001) but does not have an effect on DNMT1 acetylation. In contrast, post-treatment with PFD (5-Aza/PFD) markedly elevated the expression of scaffolding proteins, PCNA and UHRF1, as well as DNMT1 and DNMT3a (*p* < 0.00001), while DNMT3b showed a modest but significant increase (*p* < 0.001). On the other hand, post-treatment with rosiglitazone did not significantly affect the expression of these proteins of interest.

The oncoproteins c-Myc and β-catenin expression patterns in the nucleus and the anticancer marker, p53, were also examined. Representative WBs are shown in Figure 5H, in which treatment with 5-Aza decreased p53 expression; however, after combined 5-Aza/PFD treatment, p53 expression is observed (5I; *p* < 0.0001). Conversely, combined treatment with 5-Aza/PFD prevented increased β-catenin (5J; *p* < 0.0001) and c-Myc (5K; *p* < 0.001) oncogenes expression induced by 5-Aza treatment. Also, rosiglitazone treatment enhanced p53 expression and reduced β-catenin and c-Myc levels in the nuclear fraction (Figure 5H). These data suggest that PFD can effectively suppress genes implicated in carcinogenesis, which reduces cell viability and, as the MTT experiment showed, lowers rates of cell proliferation (Supplementary Figure S1). 

To corroborate the effect of PFD on DNMTs, we performed an immunofluorescence assay. Figure 5L shows in the green channel the nuclear expression of DNMT1, DNMT3a, and DNMT3b in cells without treatment and in cells treated with PFD for 48 h. The signal intensity decreased significantly in cells incubated with 5-Aza for 48 h, comparable to the red signal of 5mC. Remarkably, in cells treated with PFD and after 5-Aza treatment, a significant increase in DNMT1, DNMT3a, and 5mC signals was observed. 

Finally, a dot blot assay of the total genomic DNA extracted from HepG2 cells with the different treatments was performed. Figure 5M,N show that 5mC spot is decreased in cells treated with 5-Aza. However, when post-treatment with PFD is performed, DNA methylation regains its basal levels.

### 3.6. In Silico Analysis Reveals That PPARγ Complexes with DNMT1

**Figure 6:**
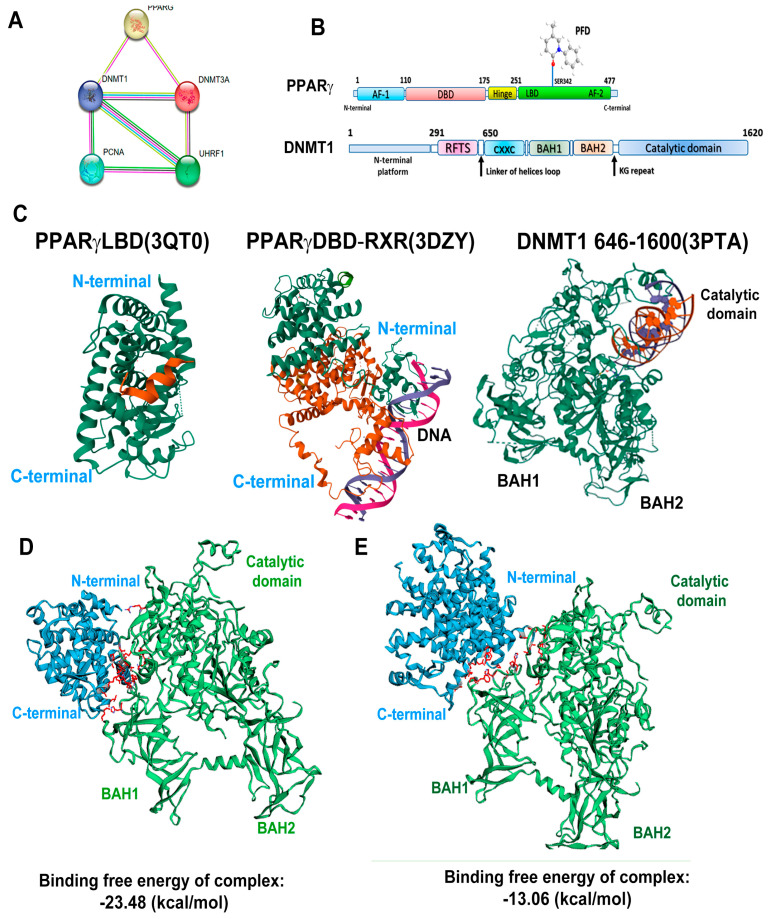
Analysis of the protein–protein interaction between PPARγ and DNMT1. (**A**) STRING database was used to generate a comprehensive protein–protein interaction (PPI) network between PPARγ and DNMT1. (**B**) The interaction between PFD and PPARγ is depicted, along with a linear representation of PPARγ and DNMT1 and their corresponding structural domains. (**C**) The 3D structures of PPARγLBD (3QT0), PPARγDBD-RXR (3DZY) and DNMT1 (3PTA) were obtained from the Protein Data Bank (https://www.rcsb.org/ (accessed on 28 May 2024)). (**D**) The high-scoring docking poses from the docking simulation of PPARγLBD-DNMT1 proteins are shown, with PPARγ (cyan blue) serving as a possible receptor and DNMT1 (green) as a possible ligand. Interacting amino acids are highlighted in red. (**E**) The docking poses from the docking simulation of PPARγDBD-RXR-DNMT1 proteins are shown, with PPARγ (cyan blue) serving as a possible receptor and DNMT1 (green) as a possible ligand. Interacting amino acids are highlighted in red.

Using the STRING platform, we performed an in silico assay to determine the possible interactions of various PPARγ isoforms with the DNMT1, PCNA, and UHRF1 proteins. Figure 6A shows that PPARγ interacts with DNMT1 and DNMT3a and indirectly with PCNA and UHRF. We also performed representative designs of the interaction between PPARγ and PFD, highlighting its interaction with the ligand binding domain (LBD). Furthermore, a second design represents the structure of DNMT1, highlighting the main domains of this protein (Figure 6B). The crystal structures for PPARγLBD (PDB:3QT0), PPARγDBD-RXR (PDB:3DZY), DNMT1 domains BAH1, BAH2, and catalytic domain (PDB:3PTA) were downloaded from the RCSB protein data bank (Figure 6C).

The HawkDock server was used to determine the interaction of both proteins. Figure 6D shows the interaction of the N-terminal of the ligan binding domain (LBD) of PPARγ with the BAH1 domain of DNMT1 with an affinity energy value of −23.48 kcal/mol, causing a change in the conformation of the catalytic domain of DNMT1. Figure 6E shows the interaction between PPARγDBD-RXR and the BAH1 domain of DNMT1 with an affinity energy value of −13.06 kcal/mol, causing a major modification of the catalytic domain of DNMT1. The above suggests that PPARg LBD exhibits greater affinity to DNMT1 without altering the catalytic domain.

Finally, the ATTRAC algorithm was used to identify the amino acids with the highest binding free energy, which was verified by MM/GBSA. Furthermore, the docking molecular models with higher binding energy are shown in Supplementary Figure S13.

## 4. Discussion

PFD is an effective and safe drug approved by the FDA for treating idiopathic pulmonary fibrosis (IPF). Also, a new formulation named prolonged release PFD (PR-PFD) was approved in 2014 by COFEPRIS in Mexico for the treatment of both IPF and Advanced Liver Fibrosis (ALF), and its use in managing liver diseases has been demonstrated. Thus, Poo et al. showed the efficacy of the administration of 1200 mg of PR-PFD dosed in 600 mg tablets twice a day for one year, in patients with advanced liver damage. They observed good tolerance to the treatment, an improvement in the quality of life of the patients, and beneficial responses such as a significant decrease in fibrosis in 35% of the patients treated with PFD, an improvement in the Child–Pugh score of the patients, and the stability of the markers of liver damage, ALT and AST [9]. This study shows that this drug has a beneficial effect on experimentally induced HCC through epigenetic modulation. It activates the PPARγ-DNMT1 axis, preventing DNA hypomethylation.

Gene expression and molecular signaling abnormalities cause HCC, a progressive liver injury that alters the histopathology and physiology of this organ. This abnormal development also involves epigenetic changes. DNA methylation is the best-studied epigenetic change and is most common in HCC and other cancers [15].

The role of PPARs in HCC is controversial. High expression of PPARα can decrease tumor cell growth by inhibiting cell division and triggering cell death through IκBα and NF-κB pathways [12,16]. Furthermore, this same overexpression is associated with longer survival periods in patients with HCC [17]. On the other hand, it has been identified that NOX1 decreases the activity of PPARα, while the absence of NOX1 promotes its expression, inhibiting endothelial cell migration and angiogenesis [18]. Our study demonstrates that PFD increases the expression of PPARα in the nucleus and cytoplasm, preventing the development of this disease.

On the other hand, Yu J et al. postulated that in mouse livers and hepatic cell lines, PPARγ suppresses tumor cell growth by reducing cell proliferation and inducing G2/M phase arrest and apoptosis, suggesting that PPARγ may act as a tumor suppressor gene [19]. However, the available evidence is not yet conclusive, nor is it entirely clear whether the ligands of this molecule promote or prevent the tumorigenic process. In different cell lines and human HCC, PPARγ overexpression is an important feature in moderately and poorly differentiated tumors [20]. Nevertheless, the mechanisms are not yet well understood; it has been suggested that antagonists blocking PPARγ activity promote cell arrest and death through apoptosis of HCC cells [21]. The PPARγ agonist pioglitazone prevents HCC and reduces macroscopic tumor nodules [22]. In this work, we determined that PFD promotes the translocation of PPARγ to the nucleus, possibly improving this pathology.

Interestingly, regarding the PPARγ2 isoform, Lee YK et al. demonstrated that its expression correlates with SREBP-1c activation and phosphorylation, leading to fat accumulation induced in pathological conditions, such as obesity and diabetes, which promote the development of HCC [23]. In our work, we observed that PPARγ2 is overexpressed in the cytoplasm and nucleus of the HCC group, which may contribute to lipid accumulation. We also observed that PFD treatment reduced the expression of PPARγ2 and the phosphorylation of SREBP-1c (Ser372), decreasing fat accumulation in the liver parenchyma generated in the HCC group (Figure 3I,J).

In our study, we observed that PFD preserves hepatic cytoarchitecture, inhibits the production and accumulation of extracellular matrix fibers, and dramatically reduces GPC3 expression. As published by Capurro et al., the non-glycosylated GPC3 protein forms a complex with Wnt-β-catenin, activating its cellular signaling and promoting the progression of HCC [24]. Likewise, Hu et al. found that in an in vitro model, curcumin treatment reduces Wnt/β-catenin signaling and GPC3 expression, while in in vivo assays, curcumin inhibits cell proliferation and apoptosis [25]. Our results show that PFD decreases the expression of GPC3 at the membrane level, blocking the translocation of β-catenin to the nucleus and decreasing the expression of the c-Myc oncogene. Recently, our research group proposed that this molecular pathway can be triggered by the interaction of PFD with PPARγ, affecting β-catenin re-localization and signaling in the HepG2 line [26].

DNA methylation is mainly carried out by DNMTs, which play a crucial role in epigenetic reprogramming. Inhibitors of these enzymes, such as 5-Aza, significantly improve the efficiency of this process, changing p53-mediated gene expression, such as apoptosis or cell proliferation [13]. Evidence suggests a regulatory link between c-Myc activity and DNMT1 and 3a; elevated levels of c-Myc have been linked to increased DNA methylation by DNMT3a [14]. Furthermore, it has been proposed that c-Myc overexpression can induce DNA damage by generating reactive oxygen species, promoting oncogenesis [13]. Although DNMT1 and p53 participate in different cellular processes, there may be interactions between various cellular pathways. It has been postulated that p53 regulates the synthesis of DNMT1 and that alterations in its function affect DNA methylation patterns. Cancer development may be related to molecular alterations through deregulation of DNMT1 and p53 [27].

Human cancers display variable patterns of DNA methylation, including gene-specific promoter hypermethylation and genome-wide hypomethylation [28,29,30,31,32]. This turns out to be undoubtedly controversial. Patients with HCC may have increased DNA methylation and overexpression of DNMT1, DNMT3a, and DNMT3b in the early stages of the disease [33,34,35]. Experimental models of early HCC show this response [36]. These results suggest that aberrant DNA methylation predicts poor disease survival. On the contrary, in normal cells, heterochromatin is highly methylated, epigenetically silencing transcriptional activity. However, global hypomethylation is observed in several types of cancer, causing genomic instability and mitotic recombination, leading to tumor development [37]. Eden A. et al. suggest that DNA hypomethylation alters chromosomal stability, favoring cancer genesis. This research group states that more profound studies are required to explain the relationship between DNA methylation and the composition and structure of chromatin to understand how hypomethylation affects DNA integrity in cancer [38].

Our results indicate that in the HCC group, the expression of DNMT1 and DNMT3a decreases, consequently causing global DNA hypomethylation. Meanwhile, PFD treatment induces the overexpression of both isoforms of DNMTs and the scaffolding protein UHRF1, which could suggest that this is the mechanism through which PFD reverses the global DNA hypomethylation caused during experimentally induced HCC. In our in vitro assay, 5-Aza administration reduces global DNA methylation and the expression of DNMT1 and DNMT3a; however, PFD reduces the effects of 5-Aza on both DNMT isoforms.

On the other hand, proliferating cell nuclear antigen (PCNA) is a nuclear protein that participates in the G1/S phases of the cell cycle and is used as a marker of cell proliferation [39]. In a study by Nishimori H et al., PCNA expression increased in patients with HCC [40]. Interestingly, in our in vivo assay, we observed that during HCC, the expression of this protein increases, while PFD prevents this response (Figure 4A). While Tikoo K. et al. postulate that incorporating 5-Aza into DNA causes direct cytotoxicity and antiproliferative effects in tumor cells [41], our in vitro results show that 5-Aza/PFD treatment maintains basal levels of PCNA. However, to fully understand the effects of PFD on PCNA, we believe that additional experiments are crucial.

Recently, Pazienza et al. demonstrated that PPARγ and DNMT1 are equally expressed in human pancreatic cancer cell lines, suggesting their importance in cancer genesis [42]. For their part, Ceccarelli et al. proposed that the activation of PPARγ by eicosatetraenoic acid improves the interaction with DNMT1 and HDAC1 in the CpG islands of the Hic-1 gene [43], while Sharma A et al., examined the PPARγ-DNMT1 interaction through PPAR-binding elements (PPRE) in an in silico model [44]. Our molecular docking analyses suggest that PFD acts as an agonist ligand of PPARγ-LBD, facilitating the formation of complexes with the BAH1 domain of DNMT1 (ΔG −23.48 kcal/mol), enhancing its methyltransferase activity.

## 5. Conclusions

**Figure 7:**
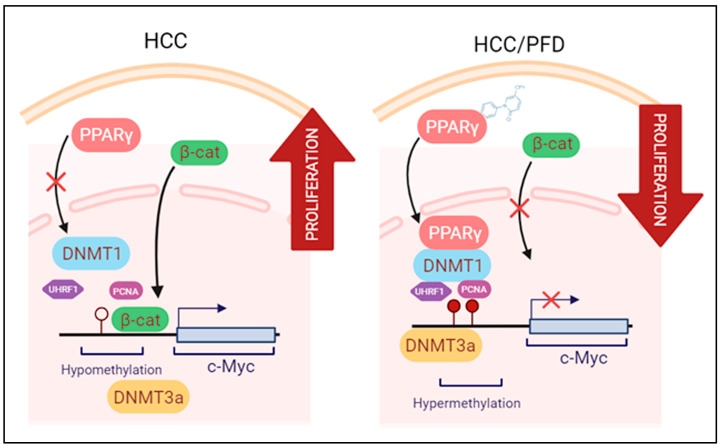
Modulation of epigenetic markers induced during HCC is the proposed mechanism exerted by PFD. Left panel: Molecular mechanisms activated during HCC growth: β-catenin crosses into the nucleus, facilitating c-Myc oncogene transcription. Right panel: PFD is a PPARγ ligand/agonist that alters DNMT1 and DNMT3a function, promoting DNA hypermethylation, and reducing c-Myc expression. These mechanisms together could suppress aberrant cell division leading to HCC.

Our results show that PFD treatment slows down the development of experimental hepatocarcinogenic damage. This effect takes place through several pharmacodynamic mechanisms including an increase in PPARγ nuclear translocation and its expression, a decrease in tumor markers expression, and, on the other hand, acting as a strong epigenetic regulator through the possible formation of PPARγ-DNMT1 complex, preventing DNA hypomethylation observed in HCC. Based on the results and the preventative nature of the experimental model used in this work, we suggest further investigation into the molecular mechanisms of PFD and its potential application as an adjuvant therapy in treating HCC. Figure 7 is a schematic representation of epigenetic mechanisms by which PFD can prevent the development of HCC.

## Figures and Tables

**Table 1:** Effects of PFD on number of hepatocellular nodules in rats.

Groups	No of Rats with Nodules/Total Rats	Nodule Incidence (%)	Total No. of Nodules	Average No. of Nodules/Nodule Bearing Liver (Nodule Multiplicity)	Nodules Relative to Size (% of Total No.)		
					≥3 mm	<3 to >1 mm	≤1 mm
NT	0/0	0	0	0	0	0	0
HCC	6/6	100	424	106 ± 36.36	12	30	58
HCC/PFD	6/6	90	227	57 ± 12.66	8	15	76

**Table 2:** Histologic alterations induced at 12 weeks.

Histological Parameter	NT	HCC	HCC/PFD
Hyperplasia	−	+	+
Dysplasia	−	+	−
Cancer cell	−	+ (MD)	+ (WD)
Cellular infiltration	−	1	0
Fibrosis	−	3	2
Oval cells	−	3	1
Ballooning degeneration	−	3	1
Steatosis	−	++	−
Cholestasis	−	0	0
Mallory bodies	−	+	−
Lobular inflammation	−	1	0
Periportal bile ducts proliferation	−	1	0

Description of histopathological scoring of the different groups: NT, non-treated; HCC, hepatocellular carcinoma group; HCC/PFD, HCC plus pirfenidone group. MD, moderate-differentiated HCC; WD, well-differentiated HCC. The scores are: 0, none, 1—mild, 2 —moderate, 3—large, +, present, ++ abundant and −, absent.

## Data Availability

Data are contained within the article and Supplementary Materials.

## Supplementary Materials

The following supporting information can be downloaded at: https://www.mdpi.com/article/10.3390/cells13121013/s1.

## References

[1] Sung H., Ferlay J., Siegel R.L., Laversanne M., Soerjomataram I., Jemal A., Bray F. (2021). Global Cancer Statistics 2020: GLOBOCAN Estimates of Incidence and Mortality Worldwide for 36 Cancers in 185 Countries. CA Cancer J. Clin..

[2] Alqahtani A., Khan Z., Alloghbi A., Ahmed T.S.S., Ashraf M., Hammouda D.M. (2019). Hepatocellular Carcinoma: Molecular Mechanisms and Targeted Therapies. Medicina.

[3] Refolo M.G., Messa C., Guerra V., Carr B.I., D’alessandro R. (2020). Inflammatory Mechanisms of HCC Development. Cancers.

[4] Fernández-Barrena M.G., Arechederra M., Colyn L., Berasain C., Avila M.A. (2020). Epigenetics in Hepatocellular Carcinoma Development and Therapy: The Tip of the Iceberg. JHEP Rep..

[5] Braghini M.R., Lo Re O., Romito I., Fernandez-Barrena M.G., Barbaro B., Pomella S., Rota R., Vinciguerra M., Avila M.A., Alisi A. (2022). Epigenetic Remodelling in Human Hepatocellular Carcinoma. J. Exp. Clin. Cancer Res..

[6] Galicia-Moreno M., Silva-Gomez J.A., Lucano-Landeros S., Santos A., Monroy-Ramirez H.C., Armendariz-Borunda J. (2021). Liver Cancer: Therapeutic Challenges and the Importance of Experimental Models. Can. J. Gastroenterol. Hepatol..

[7] Castro-Gil M.P., Sánchez-Rodríguez R., Torres-Mena J.E., López-Torres C.D., Quintanar-Jurado V., Gabiño-López N.B., Villa-Treviño S., del-Pozo-Jauner L., Arellanes-Robledo J., Pérez-Carreón J.I. (2021). Enrichment of Progenitor Cells by 2-Acetylaminofluorene Accelerates Liver Carcinogenesis Induced by Diethylnitrosamine in Vivo. Mol. Carcinog..

[8] Lopez-de la Mora D.A., Sanchez-Roque C., Montoya-Buelna M., Sanchez-Enriquez S., Lucano-Landeros S., Macias-Barragan J., Armendariz-Borunda J. (2015). Role and New Insights of Pirfenidone in Fibrotic Diseases. Int. J. Med. Sci..

[9] Poo J.L., Torre A., Aguilar-Ramírez J.R., Cruz M., Mejía-Cuán L., Cerda E., Velázquez A., Patiño A., Ramírez-Castillo C., Cisneros L. (2020). Benefits of prolonged-release pirfenidone plus standard of care treatment in patients with advanced liver fibrosis: PROMETEO study. Hepatol. Int..

[10] Zou W.J., Huang Z., Jiang T.P., Shen Y.P., Zhao A.S., Zhou S., Zhang S. (2017). Pirfenidone Inhibits Proliferation and Promotes Apoptosis of Hepatocellular Carcinoma Cells by Inhibiting the Wnt/β-Catenin Signaling Pathway. Med. Sci. Monit..

[11] Sandoval-Rodriguez A., Monroy-Ramirez H.C., Meza-Rios A., Garcia-Bañuelos J., Vera-Cruz J., Gutiérrez-Cuevas J., Silva-Gomez J., Staels B., Dominguez-Rosales J., Galicia-Moreno M. (2020). Pirfenidone Is an Agonistic Ligand for PPARα and Improves NASH by Activation of SIRT1/LKB1/PAMPK. Hepatol. Commun..

[12] Silva-Gomez J.A., Galicia-Moreno M., Sandoval-Rodriguez A., Miranda-Roblero H.O., Lucano-Landeros S., Santos A., Monroy-Ramirez H.C., Armendariz-Borunda J. (2021). Hepatocarcinogenesis Prevention by Pirfenidone Is PPARγ Mediated and Involves Modification of Nuclear NF-κB p65/p50 Ratio. Int. J. Mol. Sci..

[13] Thorgeirsson S.S., Grisham J.W. (2002). Molecular Pathogenesis of Human Hepatocellular Carcinoma. Nat. Genet..

[14] Weng G., Wang E., Wang Z., Liu H., Zhu F., Li D., Hou T. (2019). HawkDock: A web server to predict and analyze the protein-protein complex based on computational docking and MM/GBSA. Nucleic Acids Res..

[15] Nishida N., Nagasaka T., Nishimura T., Ikai I., Richard C., Goel A. (2008). Aberrant methylation of multiple tumor suppressor genes in aging liver, chronic hepatitis, and hepatocellular carcinoma. Hepatology.

[16] Zhang N., Chu E.S., Zhang J., Li X., Liang Q., Chen J., Chen M., Teoh N., Farrell G., Sung J.J. (2014). Peroxisome proliferator activated receptor alpha inhibits hepatocarcinogenesis through mediating NF-κB signaling pathway. Oncotarget.

[17] Xiao Y.B., Cai S.H., Liu L.L., Yang X., Yun J.P. (2018). Decreased expression of peroxisome proliferator-activated receptor alpha indicates unfavorable outcomes in hepatocellular carcinoma. Cancer Manag. Res..

[18] Garrido-Urbani S., Jemelin S., Deffert C., Carnesecchi S., Basset O., Szyndralewiez C., Heitz F., Page P., Montet X., Michalik L. (2011). Targeting vascular NADPH oxidase 1 blocks tumor angiogenesis through a PPARα mediated mechanism. PLoS ONE.

[19] Yu J., Shen B., Chu E.S.H., Teoh N., Cheung K.F., Wu C.W., Wang S., Lam C.N.Y., Feng H., Zhao J. (2010). Inhibitory Role of Peroxisome Proliferator-Activated Receptor Gamma in Hepatocarcinogenesis in Mice and in Vitro. Hepatology.

[20] Schaefer K.L., Wada K., Takahashi H., Matsuhashi N., Ohnishi S., Wolfe M.M., Turner J.R., Nakajima A., Borkan S.C., Saubermann L.J. (2005). Peroxisome Proliferator-Activated Receptor γ Inhibition Prevents Adhesion to the Extracellular Matrix and Induces Anoikis in Hepatocellular Carcinoma Cells. Cancer Res..

[21] Koga H., Sakisaka S., Harada M., Takagi T., Hanada S., Taniguchi E., Kawaguchi T., Sasatomi K., Kimura R., Hashimoto O. (2001). Involvement of p21WAF1/Cip1, p27Kip1, and p18INK4c in Troglitazone-Induced Cell-Cycle Arrest in Human Hepatoma Cell Lines. Hepatology.

[22] Li S., Ghoshal S., Sojoodi M., Arora G., Masia R., Erstad D.J., Lanuti M., Hoshida Y., Baumert T.F., Tanabe K.K. (2019). Pioglitazone Reduces Hepatocellular Carcinoma Development in Two Rodent Models of Cirrhosis. J. Gastrointest. Surg..

[23] Lee Y.K., Park J.E., Lee M., Hardwick J.P. (2018). Hepatic Lipid Homeostasis by Peroxisome Proliferator-Activated Receptor Gamma 2. Liver Res..

[24] Capurro M.I., Xiang Y.Y., Lobe C., Filmus J. (2005). Glypican-3 Promotes the Growth of Hepatocellular Carcinoma by Stimulating Canonical Wnt Signaling. Cancer Res..

[25] Hu P., Ke C., Guo X., Ren P., Tong Y., Luo S., He Y., Wei Z., Cheng B., Li R. (2019). Both Glypican-3/Wnt/β-Catenin Signaling Pathway and Autophagy Contributed to the Inhibitory Effect of Curcumin on Hepatocellular Carcinoma. Dig. Liver Dis..

[26] Monroy-Ramírez H.C., Silva-Gómez J.A., Galicia-Moreno M., Santos-García A., Armendáriz-Borunda J. (2020). Analysis of the Molecular Interaction of Pirfenidone with PPAR-Gamma and Effects on the Beta-Catenine Pathway in HEPG2 Line. Ann. Hepatol..

[27] Georgia S., Kanji M., Bhushan A. (2013). DNMT1 Represses P53 to Maintain Progenitor Cell Survival during Pancreatic Organogenesis. Genes Dev..

[28] Feinberg A.P. (2004). The Epigenetics of Cancer Etiology. Semin. Cancer Biol..

[29] Peter A.J., Baylin S.B. (2007). The Epigenomic of Cancer. Cell.

[30] Baylin S.B., Jones P.A. (2016). Epigenetic Determinants of Cancer. Cold Spring Harb. Perspect. Biol..

[31] Ehrlich M. (2009). DNA Hypomethylation in Cancer Cells. Epigenomics.

[32] Zhang Y.J., Wu H.C., Yazici H., Yu M.W., Lee P.H., Santella R.M. (2012). Global Hypomethylation in Hepatocellular Carcinoma and Its Relationship to Aflatoxin B1 Exposure. World J. Hepatol..

[33] Oh B., Kim H., Park H., Shim Y., Choi J., Park C., Park Y.N. (2007). DNA methyltransferase expression and DNA methylation in human hepatocellular carcinoma and their clinicopathological correlation. Int. J. Mol. Med..

[34] Arai E., Kanai Y. (2010). DNA Methylation Profiles in Precancerous Tissue and Cancers: Carcinogenetic Risk Estimation and Prognostication Based on DNA Methylation Status. Epigenomics.

[35] Pascale R.M., Simile M.M., Satta G., Seddaiu M.A., Daino L., Pinna G., Vinci Gaspa M.A.L., Feo F. (1991). Comparative Effects of L-Methionine, S-Adenosyl-L-Methionine and 5’-Methylthioadenosine on the Growth of Preneoplastic Lesions and DNA Methylation in Rat Liver during the Early Stages of Hepatocarcinogenesis. Anticancer. Res..

[36] Valencia Antúnez C.A., Chayeb L.T., Rodriguez-Segura M.Á., López Álvarez G.S., Garcia-Cuéllar C.M., Treviño S.V. (2014). DNA Methyltransferases 3a and 3b Are Differentially Expressed in the Early Stages of a Rat Liver Carcinogenesis Model. Oncol. Rep..

[37] Cheung H.H., Lee T.L., Rennert O.M., Chan W.Y. (2009). DNA methylation of cancer genome. Birth Defects Res. C Embryo. Today..

[38] Eden A., Gaudet F., Waghmare A., Jaenisch R. (2003). Chromosomal Instability and Tumors Promoted by DNA Hypomethylation. Science.

[39] González-Magaña A., Blanco F.J. (2020). Human PCNA Structure, Function and Interactions. Biomolecules.

[40] Nishimori H., Tsukishiro T., Nambu S., Okada K., Shimizu Y., Miyabayashi C., Higuchi K., Watanabe A. (1994). Analyses of proliferating cell nuclear antigen-positive cells in hepatocellular carcinoma: Comparisons with clinical findings. J. Gastroenterol. Hepatol..

[41] Tikoo K., Ali I.Y., Gupta J., Gupta C. (2009). 5-Azacytidine prevents cisplatin induced nephrotoxicity and potentiates anticancer activity of cisplatin by involving inhibition of metallothionein, pAKT and DNMT1 expression in chemical induced cancer rats. Toxicol. Lett..

[42] Pazienza V., Tavano F., Benegiamo G., Vinciguerra M., Burbaci F.P., Copetti M., Di Mola F.F., Andriulli A., Di Sebastiano P. (2012). Correlations among PPARγ, DNMT1, and DNMT3B Expression Levels and Pancreatic Cancer. PPAR Res..

[43] Ceccarelli V., Ronchetti S., Marchetti M.C., Calvitti M., Riccardi C., Grignani F., Vecchini A. (2020). Molecular Mechanisms Underlying Eicosapentaenoic Acid Inhibition of HDAC1 and DNMT Expression and Activity in Carcinoma Cells. Biochim. Biophys. Acta-Gene Regul. Mech..

[44] Sharma A., Tobar-Tosse F., Dakal T.C., Liu H., Biswas A., Menon A., Paruchuri A., Katsonis P., Lichtarge O., Gromiha M.M. (2021). PPAR-Responsive Elements Enriched with Alu Repeats May Contribute to Distinctive PPARγ–DNMT1 Interactions in the Genome. Cancers.

