# Supplementary material for: Pirfenidone Reverts Global DNA Hypomethylation, Promoting DNMT1/UHRF/PCNA Coupling Complex in Experimental Hepatocarcinoma
Source: Cells. 2024 Jun 10;13(12):1013. doi: 10.3390/cells13121013 (PMC11201610; doi:10.3390/cells13121013)
Supplement: Supplementary file 1 [file cells-13-01013-s001.zip › cells-2948636-supplementary.pdf]

Pirfenidone reverts global DNA hypomethylation, promoting DNMT1/UHRF/PCNA coupling complex in experimental hepatocarcinoma.

Hipólito Otoniel Miranda-Roblero [1,2,‡], Liliana Faridi Saavedra-Salazar [1,2,‡], Marina Galicia-Moreno [2], Scarlet Arceo-Orozco [2], Fernando Caloca-Camarena [2], Ana Sandoval-Rodriguez [2], Jesús García-Bañuelos [2], Claudia Frias-Gonzalez [2], Mónica Almeida-López [3], Erika Martínez-López [4], Juan Armendariz-Borunda [2,5] * and Hugo Christian Monroy-Ramirez [2]*

1 Programa de Doctorado en Ciencias en Biología Molecular en Medicina, CUCS, University of Guadalajara, Guadalajara 44340, Mexico
2 Institute of Molecular Biology in Medicine and Gene Therapy, Department of Molecular and Genomic Biology, University Center of Health Sciences, University of Guadalajara, Guadalajara 44100, Mexico.
3 Health Sciences University Center, University of Guadalajara, Guadalajara 44340, Mexico.
4 Institute of Translational Nutrigenetics and Nutrigenomics, Department of Molecular and Genomic Biology, University Center of Health Sciences, University of Guadalajara, Guadalajara 44100, Mexico.
5 Tecnologico de Monterrey, EMCS, Zapopan 45138, Mexico

Keywords
Hepatocellular carcinoma, DNMT1, DNMT3a, c-Myc, beta-catenin

*Corresponding authors:

Juan Armendariz-Borunda and Hugo Christian Monroy-Ramirez

University of Guadalajara, Institute of Molecular Biology in Medicine and Gene Therapy, CUCS.
Sierra Mojada 950, Col. Independencia.
C.P. 44340, Guadalajara, Jalisco, México.
Phone: +52 (33) 1058 5200, ext 33882.
Email: armdbo@gmail.com, hugo.monroyram@academicos.udg.mx

[1] Hipólito Otoniel Miranda-Roblero and Liliana Faridi Saavedra-Salazar are co-first authors

Supplementary data

Supplementary Table S1. Antibodies list employed				
**Antibody**	**Epitope**	**Host- class**	**Procedure**	**Reference provider**
PPARα	PPR-alpha 1-98	Mouse monoclonal	WB	Cell Signaling
PPARɣ	PPARɣ Ser 112	Rabbit IgG	WB	Cell Signaling
PPARɣ2	PPARɣ2 1-30	Mouse monoclonal	WB	Santa Cruz Biotechnology
pSREBP	Phospho-SREBP-1c (Ser372)	Rabbit IgG	WB	Cell Signaling
DNMT1 Ac	DNMT1Ac (K1127/K1129/K1131/K1133)	Rabbit IgG	WB	Biorbyt
DNMT1	DNMT1	Mouse monoclonal	WB/IF	Santa Cruz Biotechnology
DNMT3B	DNMT3B	Rabbit polyclonal	WB/IF	Santa Cruz Biotechnology
DNMT3A	DNMT3A 457-486	Rabbit Ig	WB/IF	Santa Cruz Biotechnology
SREBP1	SREBP1	Rabbit IgG	WB	Cell Signaling
UHRF1	UHRF1	Rabbit IgG	WB	Santa Cruz Biotechnology
PCNA	PCNA	Mouse monoclonal	WB	Santa Cruz Biotechnology
β-Catenin	β-Catenin 755-781	Mouse monoclonal	WB	Santa Cruz Biotechnology
C-Myc	C-Myc 408-493	Mouse monoclonal	WB	Santa Cruz Biotechnology
Lamin-B1	Lamin-B1 559-586	Mouse monoclonal	WB	Santa Cruz Biotechnology
5-mC	5-mC	Mouse monoclonal	DB/IF	Genetex
β-Actin	β-Actin 1-100	Mouse monoclonal	WB	Abcam

WB: Western blot, **IF**: Immunofluorescence, **DB**: Dot blot

Supplementary materials and methods

Biochemical determination of ɣ-GTP and ALT

For the evaluation of liver function, the recovered blood was allowed to settle for 15-20 min and then centrifuged at 3000 rpm for 10 min at room temperature. The activity of the enzymes gamma-glutamyl transpeptidase (γ-GTP) and alanine aminotransferase (ALT) was measured. γ-GTP activity assay was performed according to the method described by Glossmann M and Neville D.M., 1972. Briefly, the transpeptidation reaction was performed at 37 °C using 10 mM gamma-glutamyl-p-nitroaniline in Tris- 200 mM HCL (pH 8.2. Bio-Rad, Hercules, CA, USA), the resulting p-nitroniline was measured spectrophotometrically at 410 nm. On the other hand, the quantification of ALT activity was performed by the conversion reaction of alanine and α-oxoglutaric acid into pyruvate, which is detectable at a wavelength of 515 nm. (Method of Reitman, S and Frankl, S.A., 1957).

MTT assay

5 x 10^3 HepG2 cells were seeded per well in 100 μl of DMEM medium with 10% serum and 5% antibiotics in 96-well plates. Treatment involved PFD, 5-AZA, and a subsequent treatment of PFD following 5-AZA, using the previously described dosages. Subsequently, 50 μl of MTT (5mg/ml) was added, removed after one hour of incubation at 37°C, followed by the addition of 100 μl of DMSO to dissolve the formazan crystals. This mixture was further incubated for 5 minutes at 37°C, after which the concentration was determined at 570 nm using a spectrophotometer.

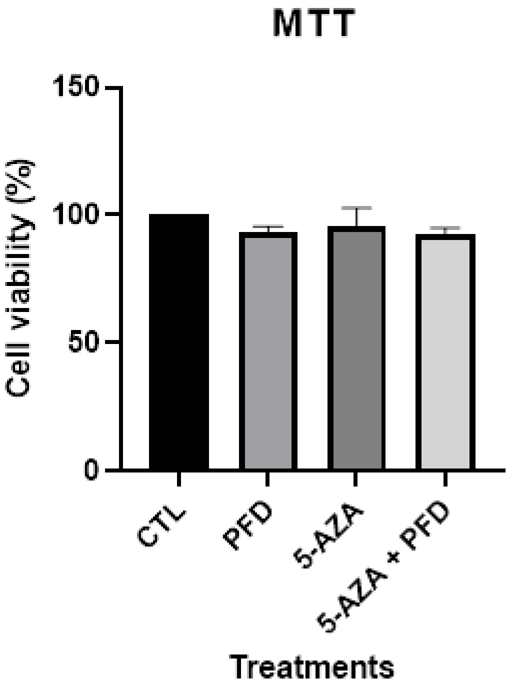

Supplementary Figure S1. Cell viability assay (MTT) was conducted using HepG2 cells treated with PFD, 5-Aza, and the combination of 5-Aza and PFD, comparing them to the viability of control cells. A trend towards decreased viability was observed in cells treated with PFD following DNMT inhibitor 5-Aza.

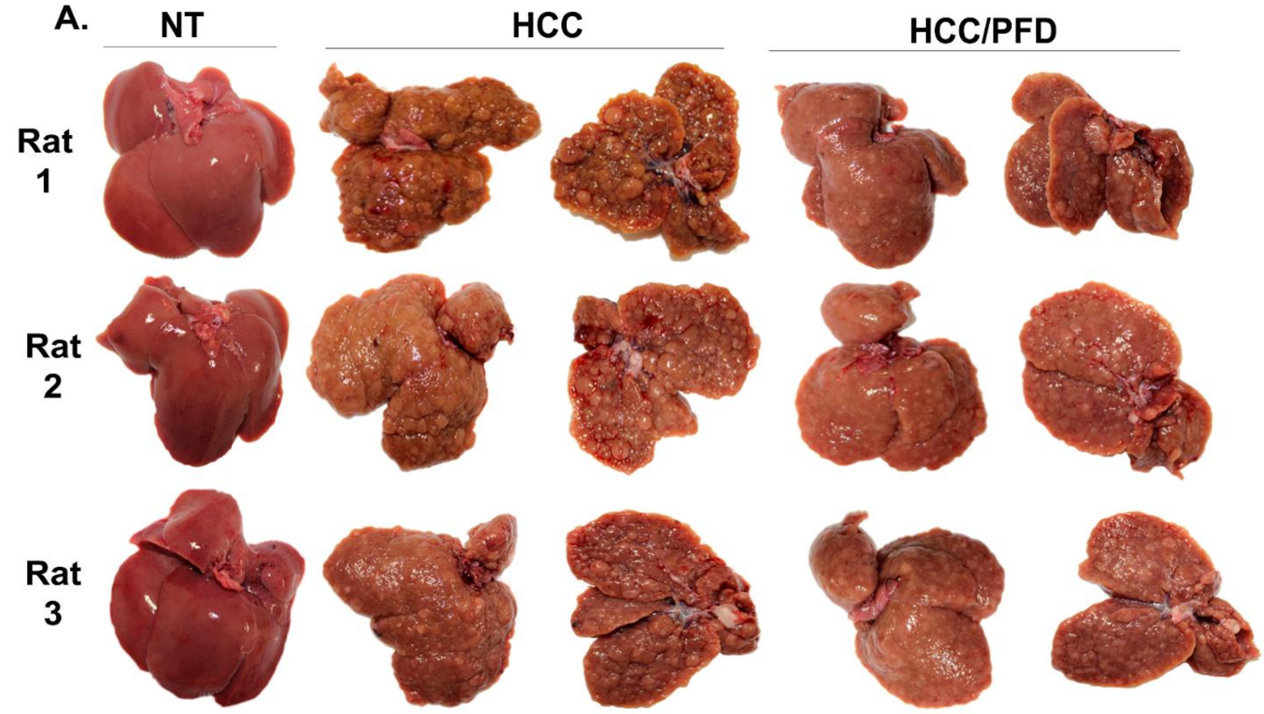

Supplementary Figure S2. Liver tissue from the NT, HCC, and HCC/PFD groups. Each group shows liver resection in triplicate. The figure shows the posterior and anterior side of the tissues, evidencing the degree of carcinogenic damage and the effect of pirfenidone on neoplastic lesions.

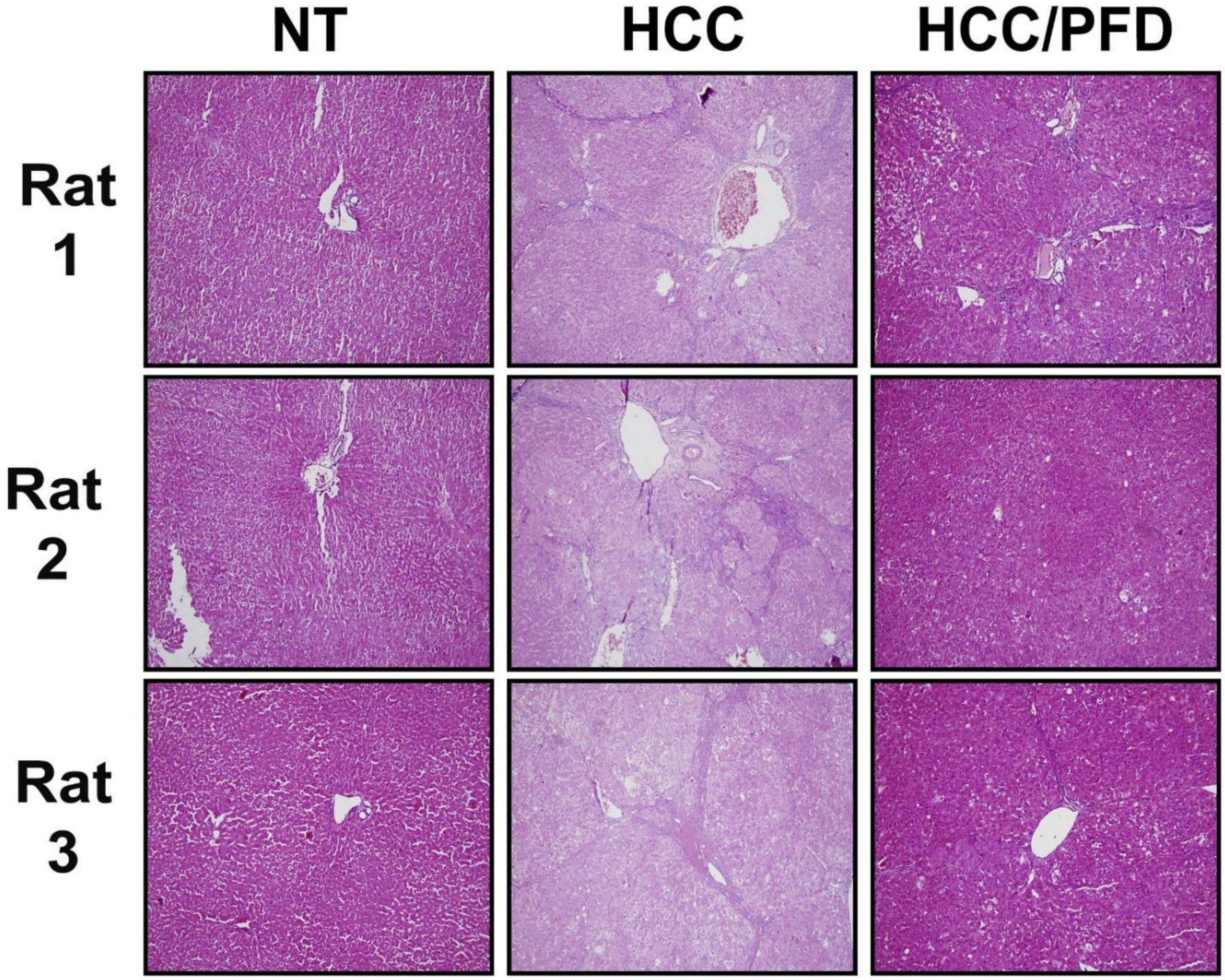

Supplementary Figure S3. Hematoxylin and eosin (H&E) staining of the NT, HCC, and HCC/PFD groups. The histological sections analyzed were performed in triplicate and were independent experiments.

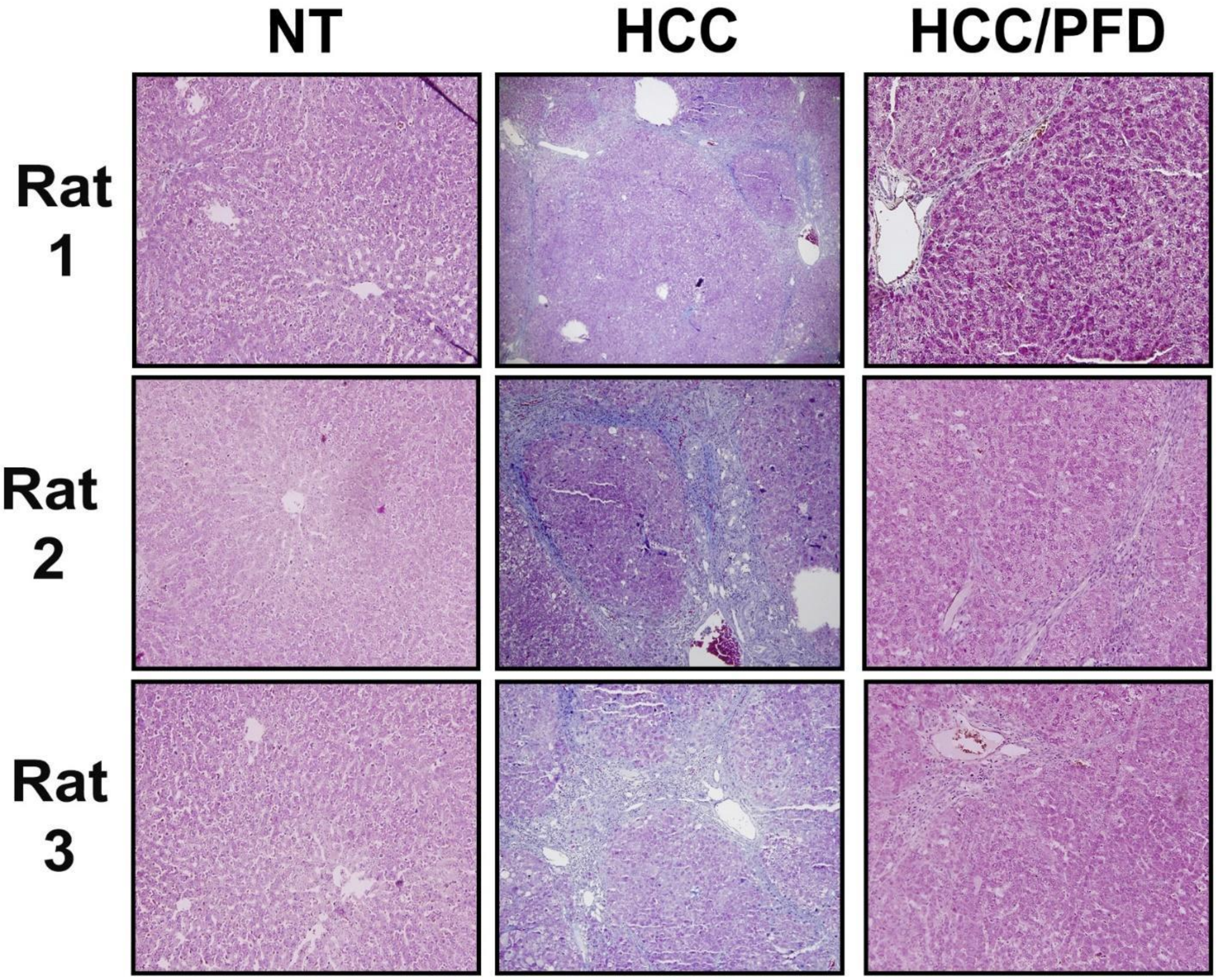

Supplementary Figure S4. Masson trichrome stain of the NT, HCC, and HCC/PFD groups. The histological sections analysed were performed in triplicate and were independent experiments.

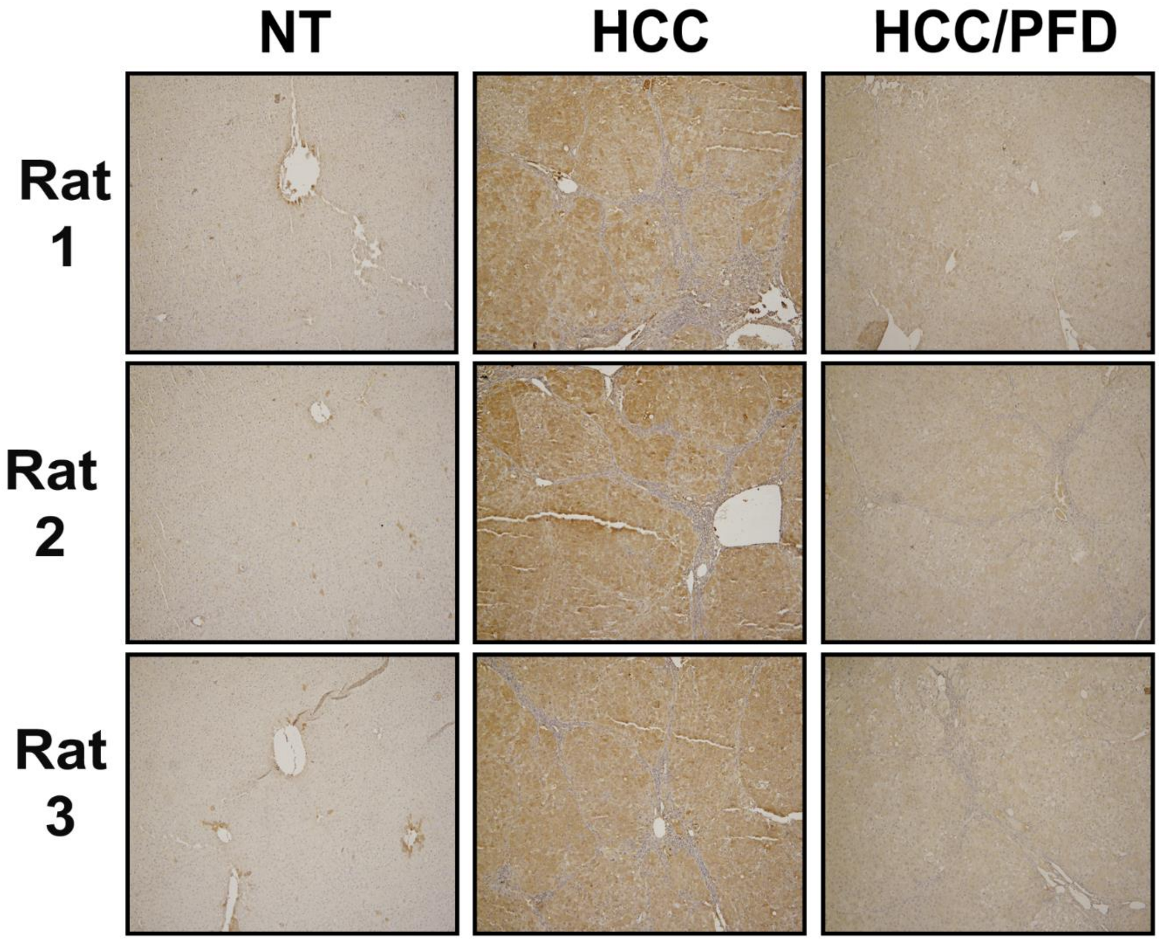

Supplementary Figure S5. Immunohistochemistry for glipican-3 (GPC3) intensity and staining pattern in liver tissue of the NT, HCC, and HCC/PFD groups. The histological sections analysed were performed in triplicate and were independent experiments.

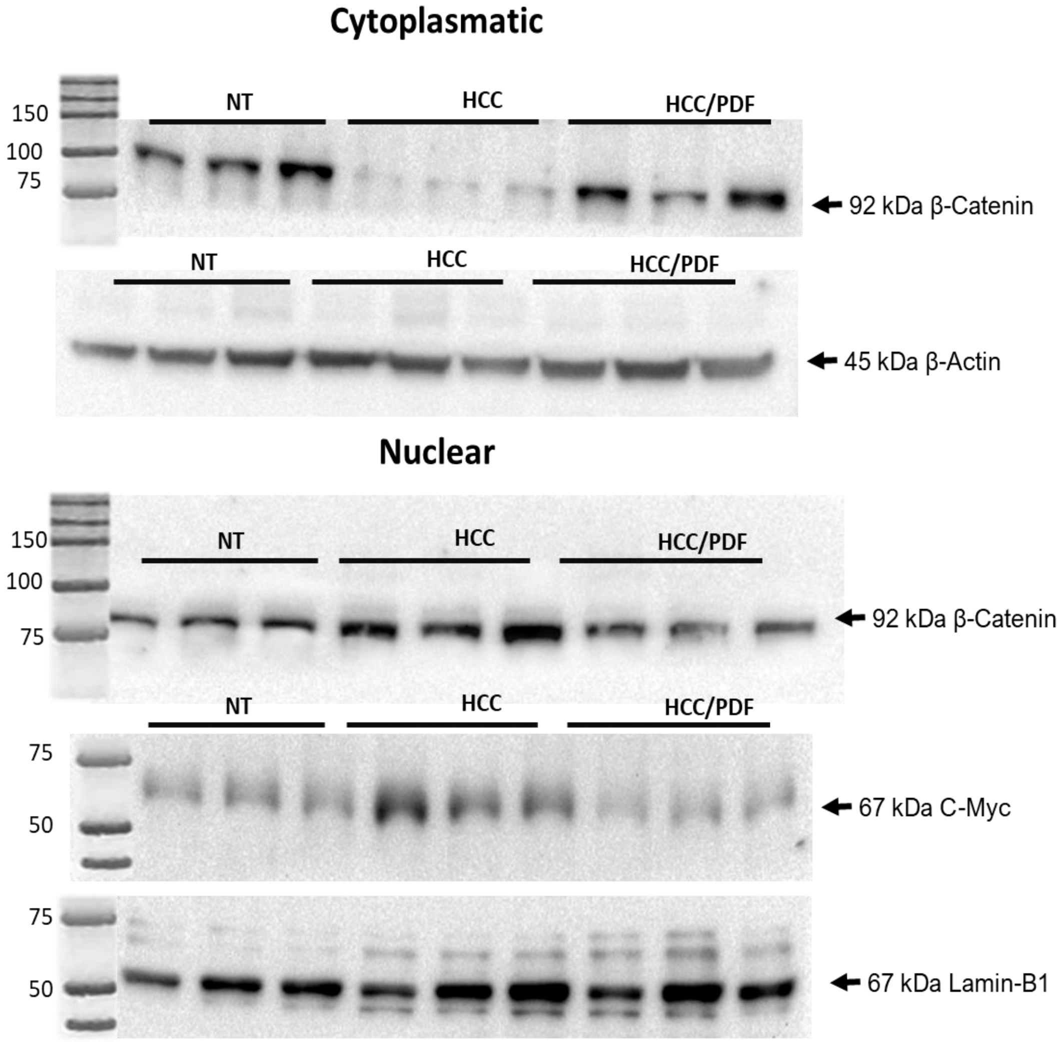

Supplementary Figure S6. Western Blot of the β-Catenin and c-Myc. The captured images show the expression bands β-Catenin and c-Myc. β-Actin was used as a loading control for cytoplasmatic extracts. Lamin-B1 was used as a loading control for nuclear extracts.

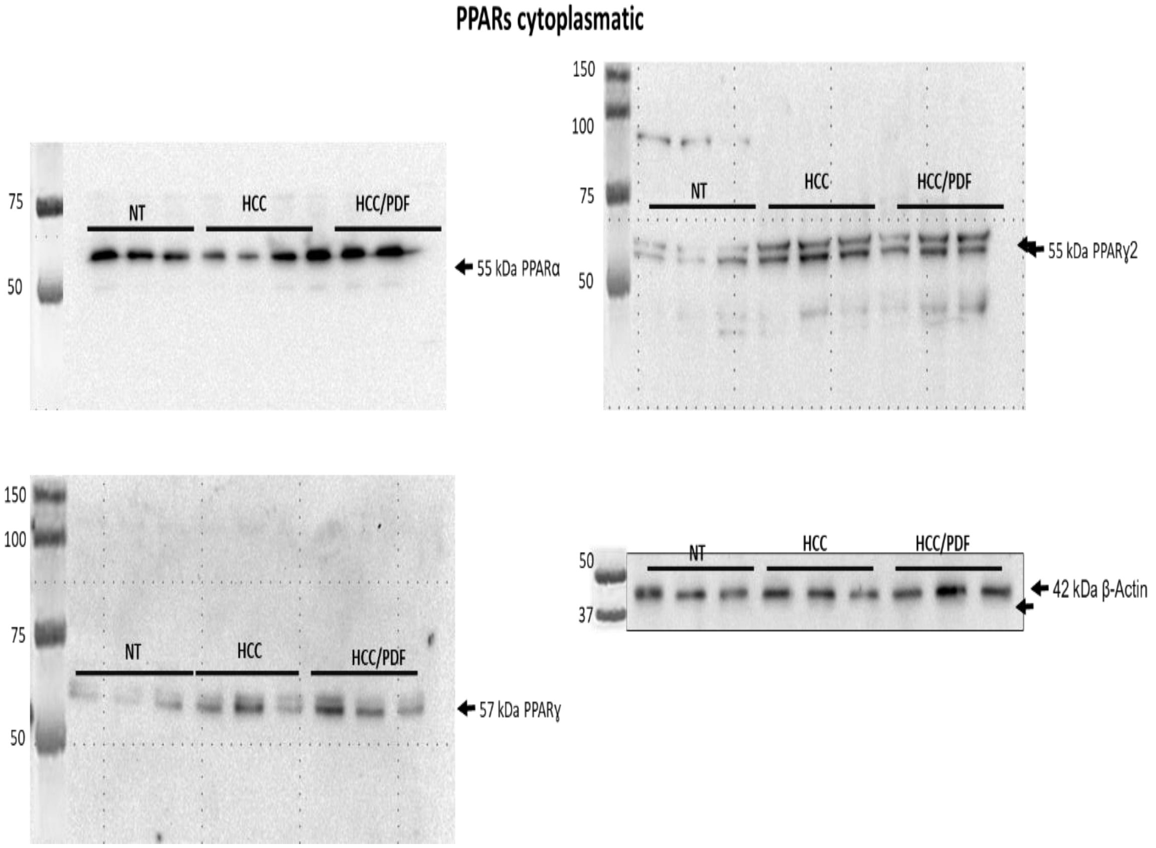

Supplementary Figure S7. Western Blot of the PPARs in cytoplasmatic fraction. The captured images show the expression bands of PPARα, PPARγand PPARγ2. β-Actin was used as a loading control for cytoplasmatic extracts.

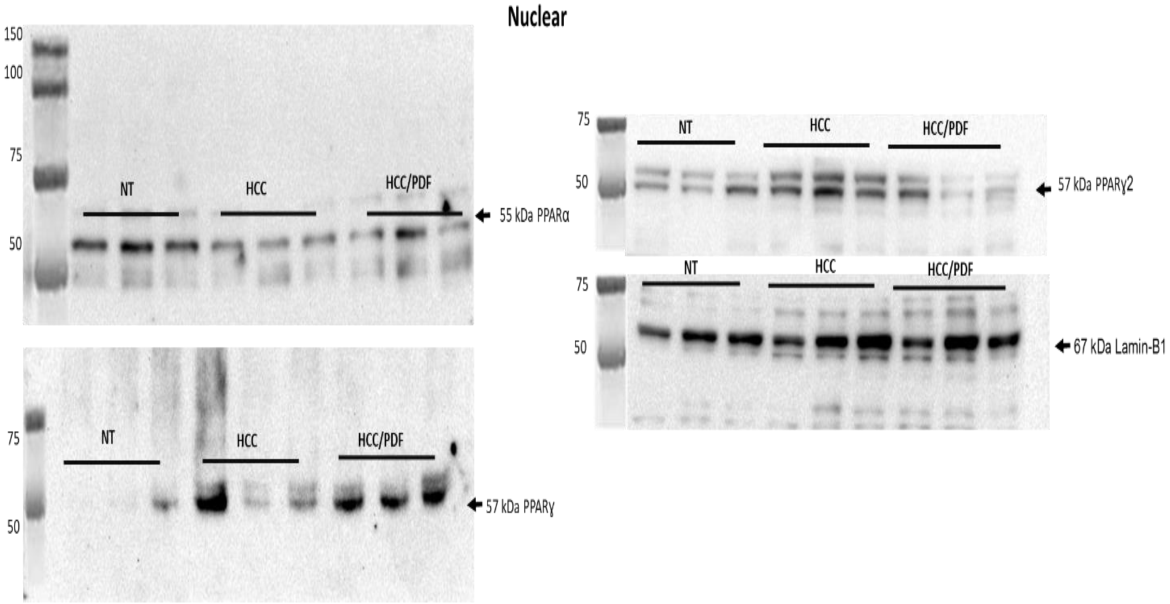

Supplementary Figure S8. Western Blot of the PPARs in nuclear fraction. The captured images show the expression bands of PPARα, PPARγand PPARγ2. Lamin-B1 was used as a loading control for nuclear extracts.

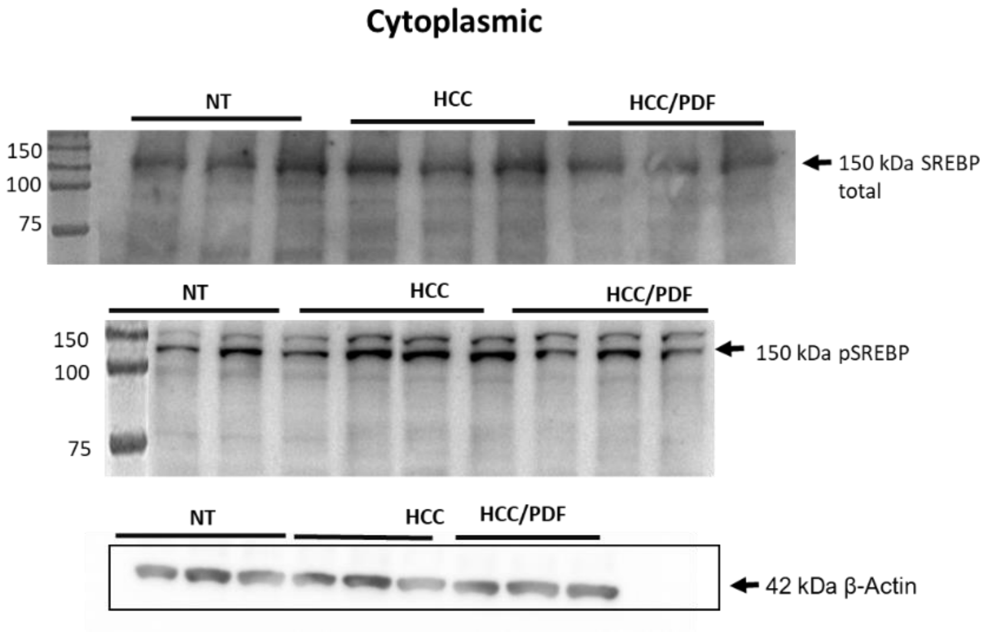

Supplementary Figure S9. Western Blot of the SREBP1 and phospo-SREBP1 in cytoplasmatic fraction. The captured images show the expression bands of SREBP1 and phospo-SREBP1. β-Actin was used as a loading control for cytoplasmic extracts.

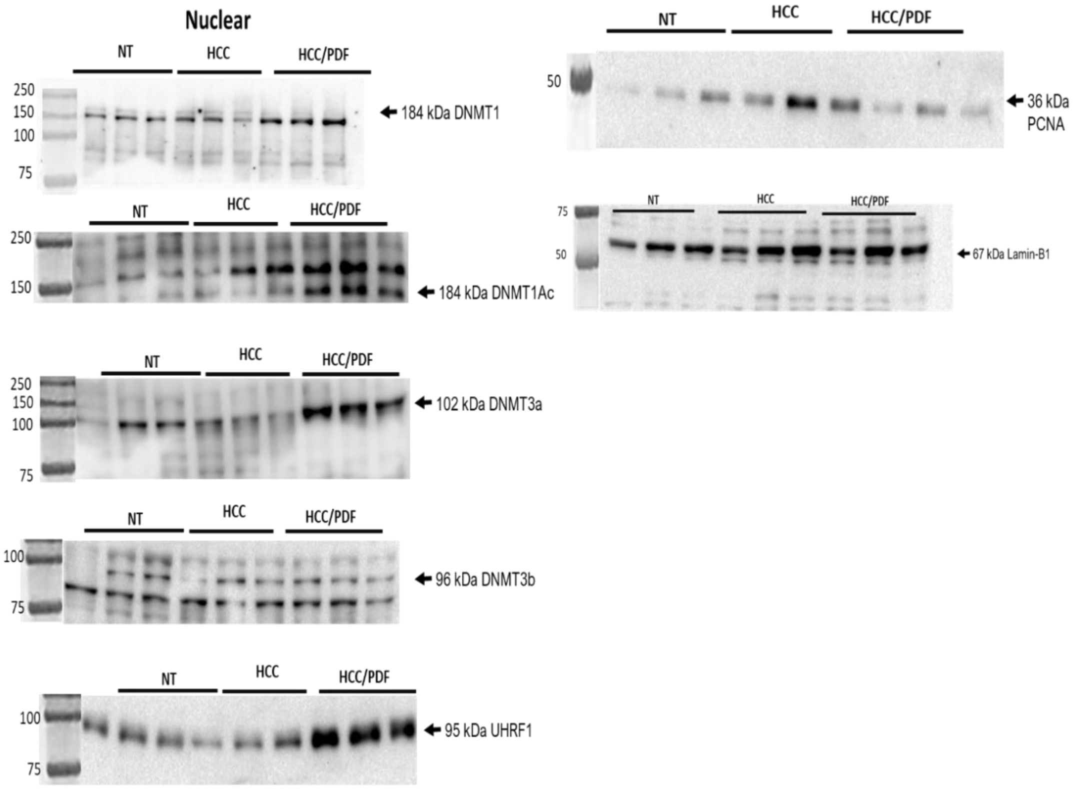

Supplementary Figure S10. Western Blot of the DNMTs isoforms and the different associated proteins. The captured images show the expression bands of DNMT1. Acetyl-DNMT1, DNMT3a, DNMT3b, UHRF1 and PCNA in nuclear extract tissues. Lamin-B1 was used as a loading control for nuclear extracts.

Nuclear western blot DNMTs, UHF1 and PCNA from HepG2

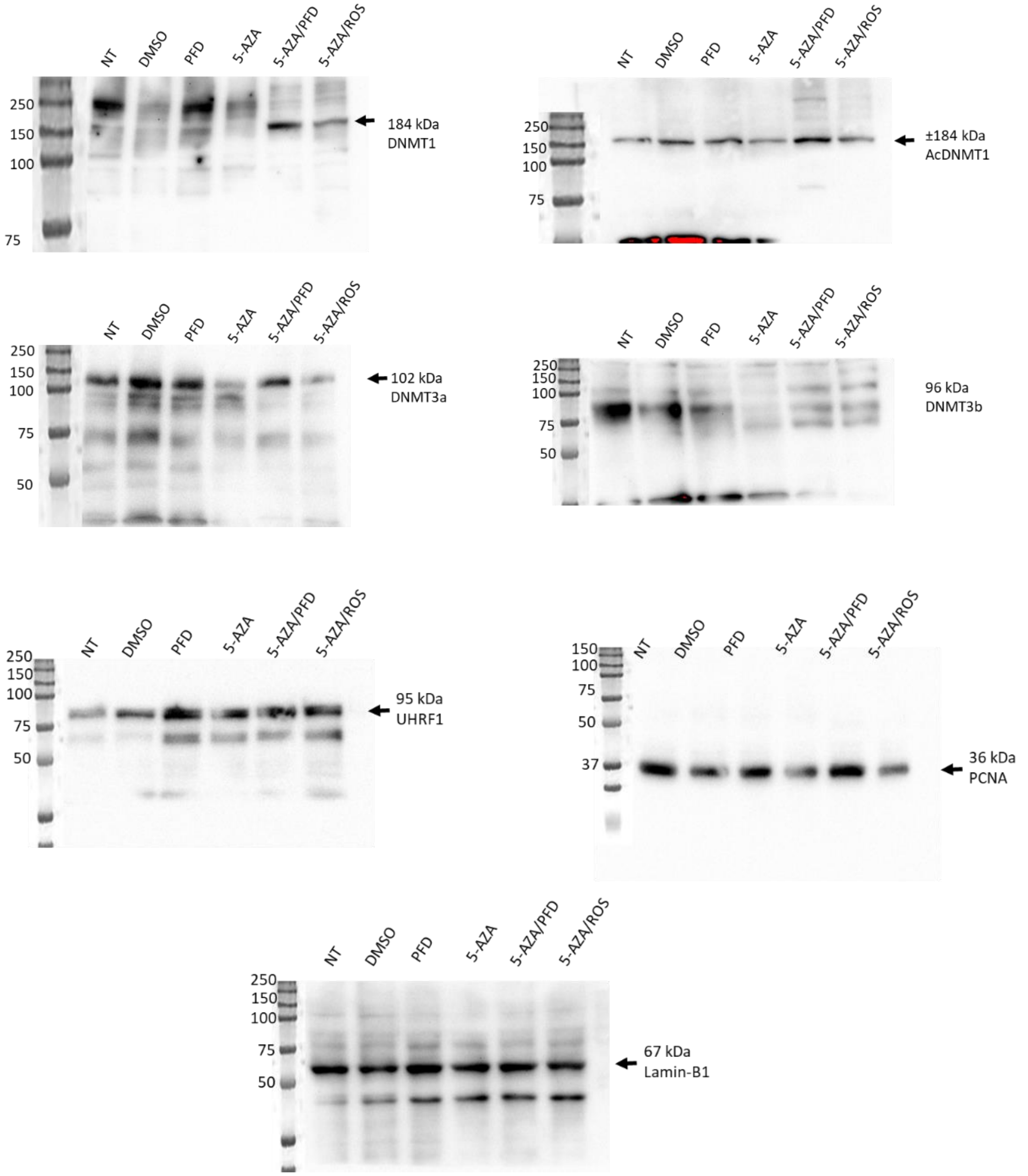

Supplementary Figure S11. Western Blot of the DNMTs isoforms and the different associated proteins. The captured images sh ow the expression bands of DNMT1. Acetyl-DNMT1, DNMT3a, DNMT3b, UHRF1 and PCNA in nuclear extract cells. Lamin-B1 was used as a loading control for nuclear extracts.

Nuclear western blot p53, β-Catenin and c-Myc from HepG2

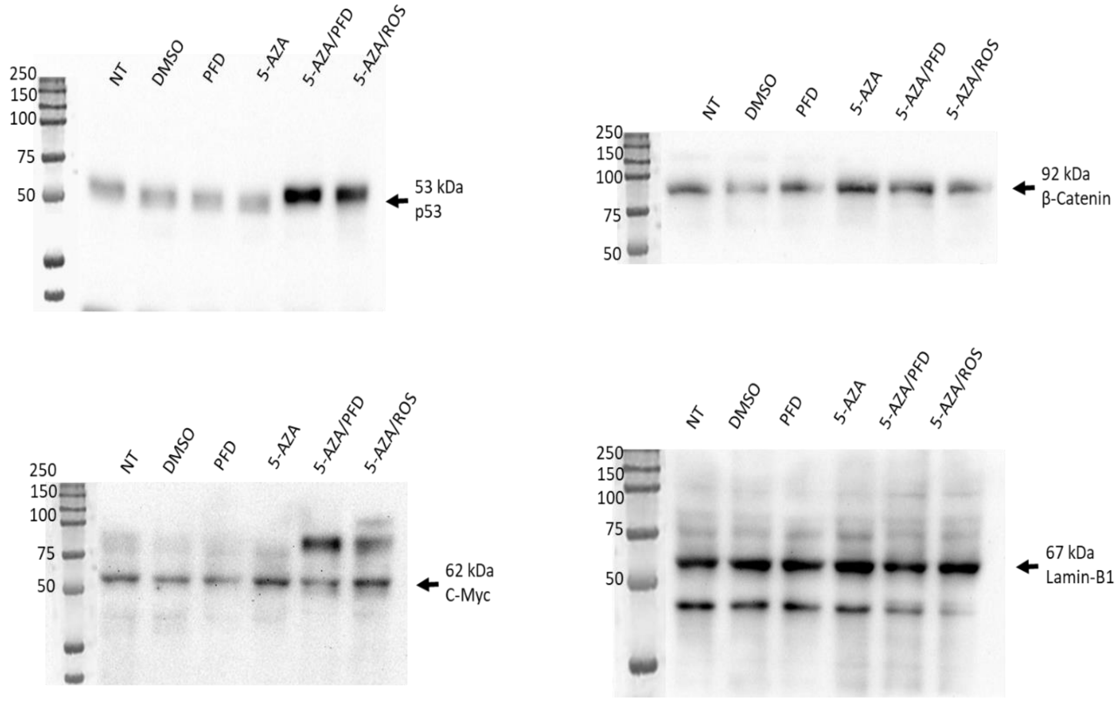

Supplementary Figure S12. Western Blot of the p53 and oncoproteins. The captured images show the expression bands of p53, β-Catenin and c-Myc. Lamin-B1 was used as a loading control for nuclear extracts.

ATTRAC algorithm was used to identify amino acids with highest binding free energy, and it was verified by means of MM/GBSA

A

PPARγLBD(3QT0)/DNMT1 (3PTA)

Rank		Residue (Rec)	Binding Free Energy
1	☐	A-ASP-14	-4.77
2	☐	A-LYS-26	-4.18
3	☐	A-LYS-34	-3.6
4	☐	A-PRO-21	-3.37
5	☐	A-ILE-17	-2.67
6	☐	A-ASP-170	-2.55
7	☐	A-LEU-213	-2.06
8	☐	A-TYR-13	-1.86
9	☐	A-ASP-4	-1.78
10	☐	A-LYS-18	-1.3

Rank		Residue (Lig)	Binding Free Energy
1	☐	B-TYR-370	-5.54
2	☐	B-HIE-371	-2.42
3	☐	B-PRO-367	-2.26
4	☐	B-LYS-302	-2.08
5	☐	B-TRP-823	-2.05
6	☐	B-ARG-686	-2
7	☐	B-ARG-676	-1.96
8	☐	B-GLN-701	-1.75
9	☐	B-HIE-363	-1.36
10	☐	B-GLY-698	-1.19

PPARγLBD(3DZY))/DNMT1(3PTA)

Rank		Residue (Rec)	Binding Free Energy
1	☐	A-ASP-433	-9.13
2	☐	A-HIE-437	-3.1
3	☐	A-ASN-436	-3.03
4	☐	A-GLU-422	-2.45
5	☐	A-PHE-431	-2.37
6	☐	A-GLU-699	-2.15
7	☐	A-PRO-435	-2.05
8	☐	A-ASP-681	-1.57
9	☐	A-GLU-382	-1.45
10	☐	A-ASP-373	-1.43

Rank		Residue (Lig)	Binding Free Energy
1	☐	B-ARG-33	-5.57
2	☐	B-LYS-29	-3.55
3	☐	B-ARG-78	-1.47
4	☐	B-SER-12	-1.41
5	☐	B-GLN-358	-1.38
6	☐	B-LYS-25	-1.36
7	☐	B-TYR-340	-1.28
8	☐	B-GLN-52	-1.25
9	☐	B-ILE-339	-1.17
10	☐	B-LEU-338	-0.82

PPARγLBD(3QT0)/DNMT1(3PTA) PPARγDBD-RXRT(3QT0)/DNMT1(3PTA)

B

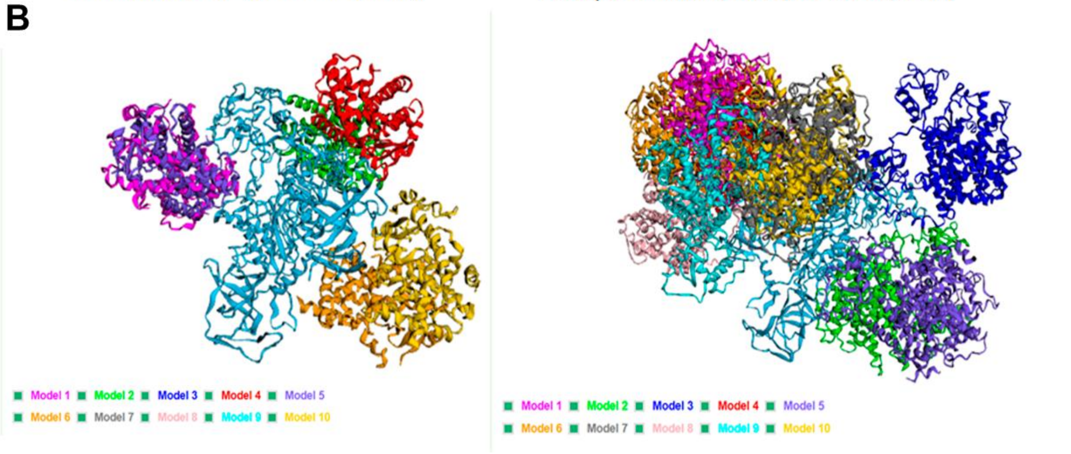

Supplementary Figure S13. A) Amino acids with highest binding free energy, and it was verified by means of MM/GBSA. B) Main molecular docking models with higher energy.
